# Caeca Microbial Variation in Broiler Chickens as a Result of Dietary Combinations Using Two Cereal Types, Supplementation of Crude Protein and Sodium Butyrate

**DOI:** 10.3389/fmicb.2020.617800

**Published:** 2021-01-11

**Authors:** Daniel Borda-Molina, Gábor Mátis, Máté Mackei, Zsuzsanna Neogrády, Korinna Huber, Jana Seifert, Amélia Camarinha-Silva

**Affiliations:** ^1^Institute of Animal Science, University of Hohenheim, Stuttgart, Germany; ^2^Division of Biochemistry, Department of Physiology and Biochemistry, University of Veterinary Medicine, Budapest, Hungary

**Keywords:** broiler chickens, microbiota, non-starch polysaccharides, butyrate, functional prediction

## Abstract

The intestinal microbiome can influence the efficiency and the health status of its host’s digestive system. Indigestible non-starch polysaccharides (NSP) serve as substrates for bacterial fermentation, resulting in short-chain fatty acids like butyrate. In broiler’s nutrition, dietary crude protein (CP) and butyrate’s presence is of particular interest for its impact on intestinal health and growth performance. In this study, we evaluated the effect on the microbial ecology of the ceca of dietary supplementations, varying the cereal type (maize and wheat), adequate levels of CP and supplementation of sodium butyrate on broiler chickens with 21 days. The overall structure of bacterial communities was statistically affected by cereal type, CP, and sodium butyrate (*p* = 0.001). Wheat in the diet promoted the presence of Lactobacillaceae, Bifidobacteriaceae and *Bacteroides xylanisolvens*, which can degrade complex carbohydrates. Maize positively affected the abundance of *Bacteroides vulgatus*. The addition of CP promoted the family Rikenellaceae, while sodium butyrate as feed supplement was positively related to the family Lachnospiraceae. Functional predictions showed an effect of the cereal type and a statistical significance across all supplementations and their corresponding interactions. The composition of diets affected the overall structure of broilers’ intestinal microbiota. The source of NSP as a substrate for bacterial fermentation had a stronger stimulus on bacterial communities than CP content or supplementation of butyrate.

## Introduction

Diet composition has a significant impact on poultry due to its influence on digestibility, gut wall morphology, and microbial structure, which might affect the health status, carcass composition and meat quality (Teirlynck et al., 2009). Maize-based (MB) diets have a higher positive impact on broilers’ performance than other cereals, which is attributed to the low presence of non-starch polysaccharides (NSP). NSPs have adverse effects on nutrient digestion and absorption (Meng et al., 2004; Lentle and Janssen, 2008; Teirlynck et al., 2009). Other cereal-based diets with higher amounts of NSPs, such as wheat, are supplemented with additive enzymes like xylanase and glucanase. This facilitates the degradation of NSPs and the release of carbon sources that promote a favorable bacterial population in the gastrointestinal tract (GIT; Lentle and Janssen, 2008). Fermentation products, like short-chain fatty acids (SCFA), are essential for the host metabolism and have positive effects on gut health.

Growth parameters in broiler chicks are also influenced by the dietary concentration of crude protein (CP), which might affect carcass yield and breast meat yield and causes a decrease in pancreas weight (Alleman et al., 2000; Lentle and Janssen, 2008; Abbasi et al., 2014). Moreover, in the intestines, an adequate protein concentration is required to maintain its viability, mass and amount of energy, where low levels resulted in the reduction of jejunal villus height and crypt depth (Abbasi et al., 2014).

The addition of butyrate in the form of sodium butyrate is seen as an alternative to promote broiler chickens’ development. This compound is solid, stable and is associated with the improvement of body weight, feed conversion ratio and the development of gut wall tissues by increasing villus height and crypt depth ratio of duodenum and jejunum (Leeson et al., 2005; Jiang et al., 2015; Ahsan et al., 2016). Furthermore, it modifies immune and inflammatory responses by decreasing the production of pro-inflammatory cytokines and mitigates the increase of corticosterone concentration, which is present during stress conditions (Zhang et al., 2011; Jiang et al., 2015). Regarding gut intestinal microbiota, butyrate is associated with increased beneficial bacterial populations while the colonization of harmful species is reduced (Ahsan et al., 2016).

To analyze the effects of dietary treatments in poultry, one of the most studied sections in the GIT is the ceca, where most fermentation processes occur, leading to the assimilation of complex substrates. This assimilation is facilitated by metabolic activities of the genera *Ruminococcus, Streptococcus, Faecalibacterium, Lactobacillus*, and *Clostridium* cluster IV, XIVa and XIVb (Borda-Molina et al., 2016; Volf et al., 2016), which colonize and maintain fermentation processes, together with the production of SCFA including butyrate.

This study aimed to describe the influence of two types of dietary cereals, two different crude protein contents, and butyrate supplementation on the cecal microbiota and its central metabolic functions in broiler chickens with 21 days.

## Materials and Methods

### Birds and Experimental Procedures

The experiment was conducted in the Research Institute for Animal Breeding, Nutrition and Meat Science at Herceghalom, Hungary. All procedures regarding animal handling and treatments were approved by the Government Office of Pest County, Food Chain Safety, Plant Protection and Soil Conservation Directorate, Budapest, Hungary (permission number: PEI/001/1430-4/2015).

A total of 240 male one-day-old Ross 308 broiler chicks were obtained from a commercial hatchery (Gallus Poultry Farming and Hatching Ltd., Devecser, Hungary) and randomly allocated to eight dietary groups (*n* = 30 per group). Specific details for experimental design have been previously published (Petrilla et al., 2018). Four of the diets consisted of maize (MB) as the cereal type and the other four were assigned to wheat [wheat-based diet (WB)] as the cereal type, supplemented with xylanase-glucanase enzyme mixture. Crude protein content was set to an appropriate dietary phase [normal protein (NP)] or reduced by 15% [low protein (LP)], the latter supplemented with essential amino acids. The feed was formulated considering the presence of sodium butyrate (But) or its absence (Ctr; Supplementary Tables 1A,B). At 21 days, 10 chickens per group were randomly selected and slaughtered by decapitation after carbon dioxide anesthesia without any starving period before sampling. Samples from cecal digesta were immediately collected and shock-frozen in liquid nitrogen and stored at −80°C until analysis.

### DNA Extraction, Illumina Amplicon Sequencing and Bioinformatic Analysis

DNA was extracted from approximately 250 mg of each digesta sample using FastDNA™ SPIN Kit for the soil from MP Biomedicals (Solon, OH, United States) following the manufacturer’s instructions. The quality and concentration of DNA were assessed through NanoDrop 2000 Spectrophotometer (Thermo Scientific, Waltham, MA, United States), and DNA was stored until use at −20°C. The V1-2 region of the 16S rRNA gene was amplified to produce Illumina sequencing library. The protocol followed the same methodology as Kaewtapee et al. (Kaewtapee et al., 2017). Briefly, 1 μl of DNA was used in a 20 μl reaction containing PrimeSTAR Hot Start DNA polymerase (2.5 U, Clontech Laboratories, Mountain View, CA, United States), 2.5 mM dNTP mixture, and 0.2 μM primers; an initial denaturation at 95°C for 3 min was followed by 20 cycles of denaturation at 98°C for 10 s, subsequent annealing at 59°C for 10 s, extension step at 72°C for 45 s and a final extension for 2 min at 72°C; 1 μl from the resultant product was taken to perform the second PCR with the aforementioned conditions in a 50 μl reaction for 15 cycles. Amplicons were verified by agarose gel electrophoresis, purified and normalized using SequalPrep Normalization Kit (Invitrogen Inc., Carlsbad, CA, United States). Samples were pooled and sequenced using 250 bp paired-end sequencing chemistry on an Illumina MiSeq platform.

Raw reads were quality filtered, assembled, and aligned using Mothur pipeline (Kozich et al., 2013). Sequences were excluded if they had any primer or barcode mismatch and an N character. A total of 5.314.942 reads were obtained, checked for chimeras using UCHIME, and clustered at 97% identity into 1,284 operative taxonomic units (OTU). Only OTUs presenting on average, an abundance higher than 0.0001% and with a sequence length > 250 bp were considered for further analysis. The closest representative was manually identified with RDP’s seqmatch function (Wang et al., 2007) and a species name was given if >97% similarity was observed with the closest representative sequence. Sequences were submitted to European Nucleotide Archive under the accession number PRJEB40780. Functional predictions were obtained with the R package Tax4Fun, which relies on the SILVA database (Quast et al., 2013). The biom table to assign this functionality was obtained from the mothur pipeline.

Correlation network analysis was done based on the SparCC algorithm (Friedman and Alm, 2012; Chong et al., 2020), making log ratio transformations and including multiple iterations to exclude taxa pairs outliers. The algorithm determines the co-abundance and co-exclusion of bacteria present in the absolute abundance (Zhang et al., 2018). The permutation was settled at 100 with a threshold value of *p* as 0.05 and a correlation threshold as 0.3 at the genus taxonomical level. Nodes indicated the genus and were colored based on their abundance for each treatment, while edges represented the correlations’ strength.

### Statistical Analysis

Datasets were analyzed with the multivariate software PRIMER (version 7.0.9, PRIMER-E, Plymouth Marine Laboratory, Plymouth, United Kingdom; Clarke and Warwick, 1994). Data were standardized by total, and similarity matrixes were created using the Bray-Curtis coefficient (Bray and Curtis, 1957). PERMANOVA analysis, using a permutation method under a reduced model, was used to study the significant differences between the dietary treatments and was considered significantly different if *p* ≤ 0.05. The community similarity structure was depicted through Principal Coordinates Analysis (PCoA). Similarity percentage (SIMPER) analysis was used to identify the OTUs responsible for the differences between the groups (Clarke and Warwick, 1994). Correlations were estimated with the Spearman coefficient using PRISM 6 (GraphPad Software, CA) and were considered significantly different if *p* < 0.05. Diversity indices (Shannon diversity and Pielou’s evenness) were calculated based on abundance data with PRIMER software.

An univariate approach within the program JMP (JMP® Pro 15.0.0, Cary, NC, United States) was used to test for individual effects on each output (OTU assignation, family assignation, or predicted function at the third KEGG level). Calculations were based on ANOVA and multiple comparisons based on the least-squares mean estimates and followed the model:

$$\begin{array}{c} y_{ijkl}=\mu +cereal\;type_{i}+crude\;protein_{j}+\\ sodium\;butyrate_{k}+\left(cereal\;type_{i}\times crude\;protein_{j}\right)+\\ \left(cereal\;type_{i}\times sodium\;butyrate_{k}\right)+\\ \left(crude\;protein_{j}\times cereal\;type_{k}\right)+\\ \left(cereal\;type_{i}\times crude\;protein_{j}\times sodium\;butyrate_{k}\right)+\\ block_{l}+e_{ijkl} \end{array}$$

## Results

Notwithstanding that the present trial was not designed to study the animals’ growth performance, it should be addressed that the growth of the broilers matched the standards of the Ross 308 breed. Body weight and average daily body weight gain data are presented in [Supplementary Table 2; according to (Petrilla et al., 2018)]. The applied dietary strategies can effectively contribute to better growth performance and carcass characteristics of broilers. The body weight of the chickens increased significantly with the low protein diet with essential amino acid supplementation during the entire trial (Supplementary Table 2).

### Taxonomical Distribution Based on Dietary Supplementations

**Figure 1:**
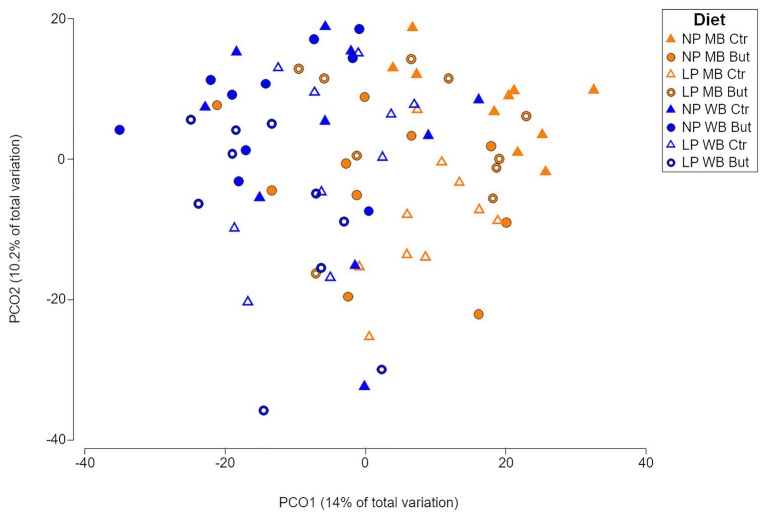
Principal coordinate analysis showing the microbial community distribution of the eight dietary treatments. NP, normal protein diet; LP, low protein diet; MB, maize based diet, WB, wheat based diet; But, sodium butyrate; Ctr, no sodium butyrate.

A significant interaction between the type of cereal, the normal or decreased content of crude protein, and the presence or absence of sodium butyrate (CeXCPxSo) was found in the caecal microbial communities (*p* = 0.001; Supplementary Table 3). Furthermore, pairwise comparisons showed that the microbial communities differed from each other in all diets (*p* ≤ 0.05; Supplementary Table 3). The sample average similarity ranged between 59% (NP MB Ctr) and 43% (LP WB But). A clustering of the caecal microbiota samples based on maize or wheat was observed sharing 54% similarity. (Figure 1 and Supplementary Figures 1, 2A). Even if significant differences were confirmed for the CP and sodium butyrate supplementation (Supplementary Table 3), there was no apparent clustering based on those criteria (Supplementary Figures 2B,C). Shannon diversity index did not show significant statistical differences between the different diets, which confirms the abundance of species as the driving factor for the variations (Supplementary Figure 3).

The most predominant phylum in all treatments was Firmicutes accounting for 57–71% of the total community and higher abundances were detected in diets with LP content (Supplementary Figure 4). Bacteroidetes was the second most abundant phylum (21–32%) with lower relative abundances in the WB diets than the maize and diets with LP contents. On the third position, Proteobacteria were 5 to 10% abundant, showing higher fractions with normal CP levels. Actinobacteria was promoted when WB diet was fed, with abundances ranging from 2 to 4% compared to 0.2–0.4% in maize-based diets. Furthermore, diets NP WB But and LP WB But, with butyrate supplementation, had more OTUs affiliated to the phylum Actinobacteria (Supplementary Figure 4).

The abundance of Bifidobacteriaceae and Lactobacillaceae increased for the WB diets (Supplementary Figure 5A). Rikenellaceae was more abundant in diets with a normal level of CP content, whereas Ruminococcaceae was promoted in the diet with low CP content (Supplementary Figure 5A and Supplementary Table 4). Additionally, sodium butyrate supplementation increased the presence of Lachnospiraceae (Supplementary Table 4). The abundance of Bacteroidaceae was affected by the interaction of CP content and sodium butyrate. This family was detected in higher abundance in the diet with normal CP and without sodium butyrate supplementation (Supplementary Table 4).

**Figure 2:**
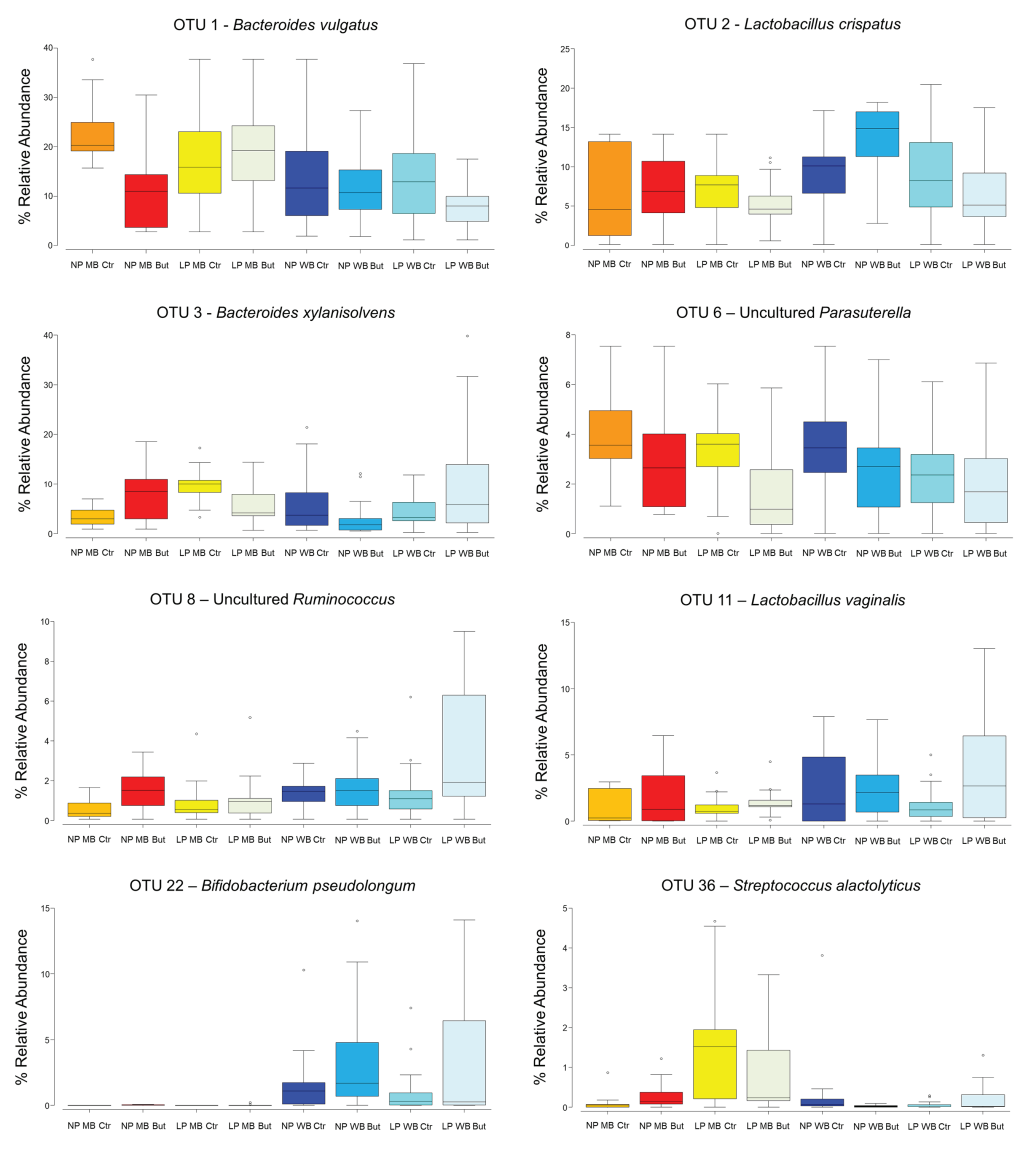
Box-plots of the most relevant operative taxonomic units (OTUs) in the eight dietary treatments.

Thirty-two OTUs were the most important contributors to the differences observed across all diets. OTU1, closely related to *Bacteroides vulgatus*, was detected in higher abundance in MB NP Ctr (23%), and in lower abundance in LP WB But (8%; Figure 2). It was significantly affected by all three factors and promoted by maize as the cereal type (*p* < 0.0001), and no sodium butyrate supplementation (*p* = 0.0008; Supplementary Table 5). OTU1 showed negative interactions with several other OTUs (Supplementary Table 6). *Bacteroides xylanisolvens* (OTU 3) was more abundant in case of diets LP WB But and LP MB Ctr (11 and 10%, respectively), whereas it was found with 4% abundance in diets NP MB Ctr, NP WB But, and LP WB Ctr (Figure 2). OTU3 was affected by the interaction of the three factors (*p* = 0.0008): maize as cereal type, low CP, and no sodium butyrate supplementation (Supplementary Table 5). OTU8 assigned to an uncultured *Ruminococcus* registered the highest abundance in LP MB Ctr (10%; Figure 2) with a tendency to be promoted by the wheat as the cereal type (*p* = 0.07) and positive correlations to other OTUs (Supplementary Table 6).

**Figure 3:**
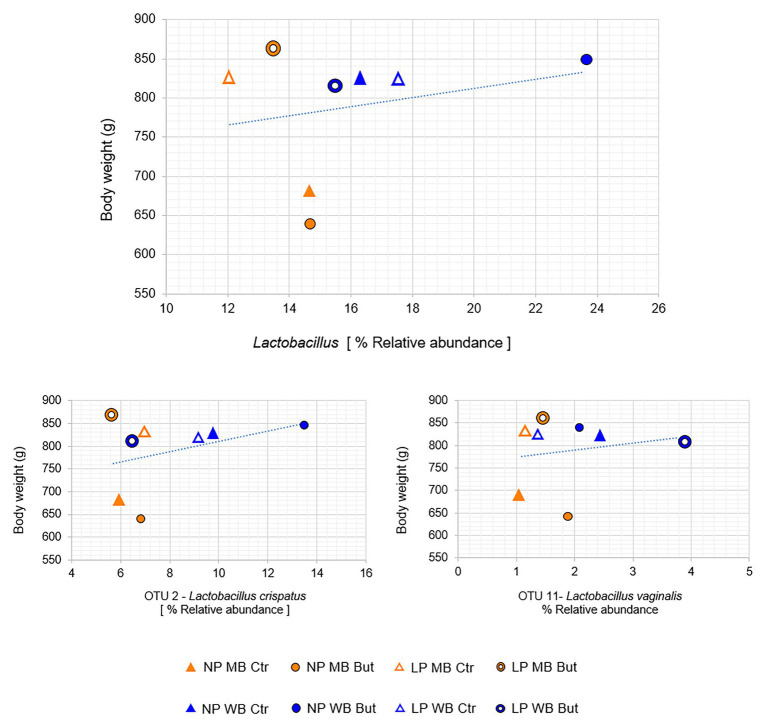
Positive correlations between body and the genus *Lactobacillus*, OTU 2 and OUT 11. (Spearman correlation resulted in positive value and *p* ≤ 0.05).

The genus *Lactobacillus*, represented by OTU2 – *Lactobacillus crispatus* and OTU11 – *Lactobacillus vaginalis*, showed a positive correlation with body weight (Figure 3). OTU2 was more abundant in WB diets such as in NP WB But (14%), while an average abundance of 6% was registered for MB diets (Figure 2). It is influenced by the interaction of crude protein level and sodium butyrate (*p* = 0.03), and a tendency was detected for the cereal type and CP (*p* = 0.07; Supplementary Table 5). OTU2 showed positive interactions with other OTUs assigned to *Lactobacillus* (Supplementary Table 6). OTU4, assigned to *Lactobacillus salivarius*, was registered in higher abundance NP MB Ctr (on average 7%) while it was detected in lower abundance in NP WB Ctr (in av. 3%). OTU4 was affected by the interaction of the three supplementations (*p* = 0.011). *L. vaginalis* (OTU11) was more abundant when wheat was used as a cereal with highest abundance in diet LP WB But (4%). This result was supported by the significant effect found with the cereal type (*p* = 0.048; Supplementary Table 5).

*Streptococcus alactolyticus* (OTU36) was 2% abundant in the LP MB Ctr diet, while the other registered percentages were lower than one. The interaction of cereal type and CP content was significant (*p* = 0.02) for this OTU. In diets with maize and wheat supplementation and normal CP (NP MB Ctr and NP WB Ctr), an uncultured *Parasutterella* (OTU6) was found in higher abundance (4%) when compared to other diets. This OTU was significantly affected by crude protein (*p* = 0.003) and sodium butyrate supplementation (*p* = 0.007; Supplementary Table 5). The cereal type had a significant effect on *Bifidobacterium pseudolongum* (OTU22; *p* = 0.0002; Supplementary Table 5) with registered abundances lower than 1% in maize cereal diets, while 1.9% (NP WB Ctr), 3.6% (NP WB But), 1.4% (LP WB Ctr), and 3.5% (LP WB But) were detected in WB diets.

**Figure 4:**
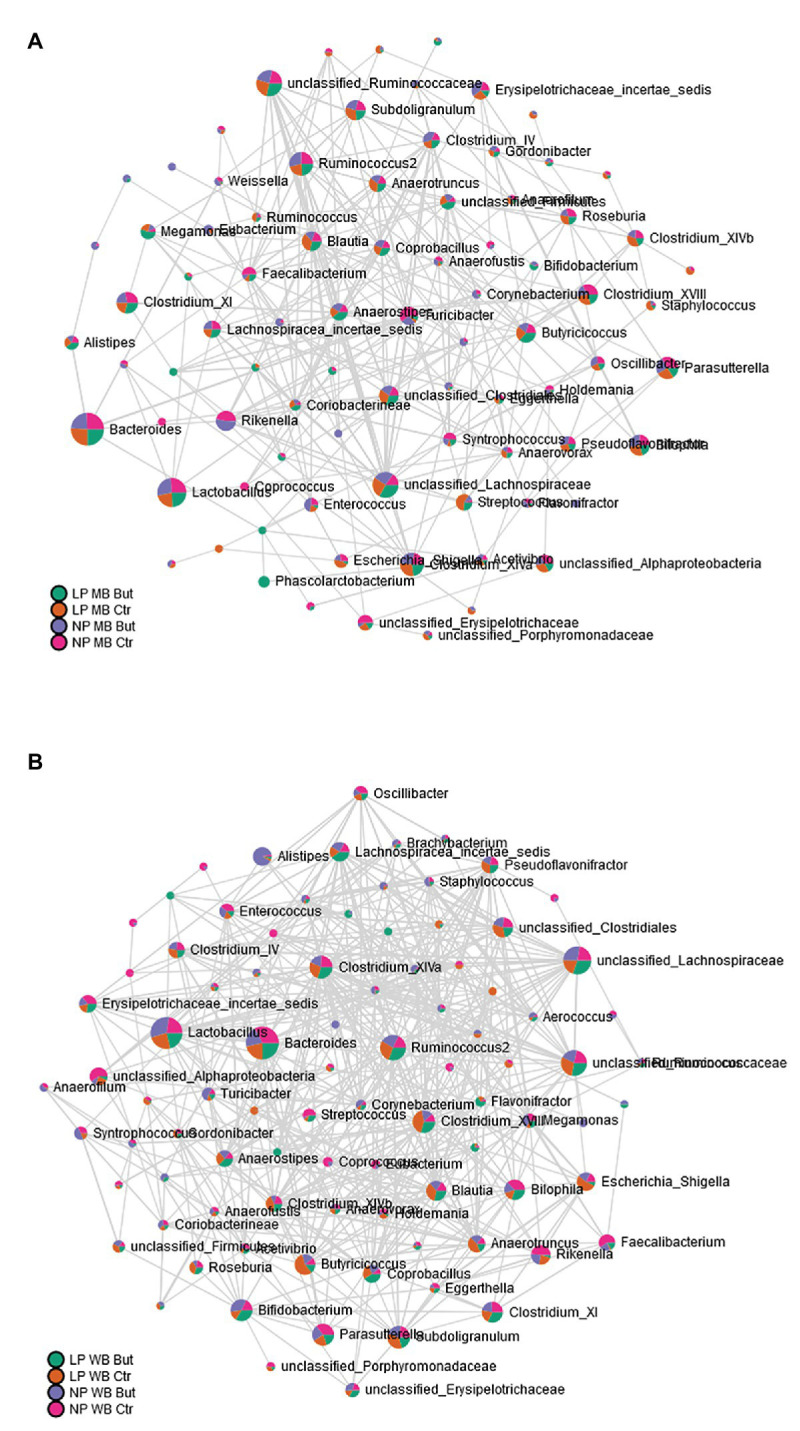
Microbial network at genus level based on correlation analysis. **(A)** MB diets and **(B)** WB diets.

The connectivity level was further inspected by a network analysis based on the differences observed between maize and wheat. The approach was followed by estimating correlation values, restricting the components to the genus level, and decreasing compositional effects. A higher quantity of significant edges was observed with wheat as a cereal type (Figure 4). More connections to the Lactobacillus and Bacteroides genera were observed compared to the MB diet.

### Functional Prediction of the Microbial Communities Influenced by the Diets

**Figure 5:**
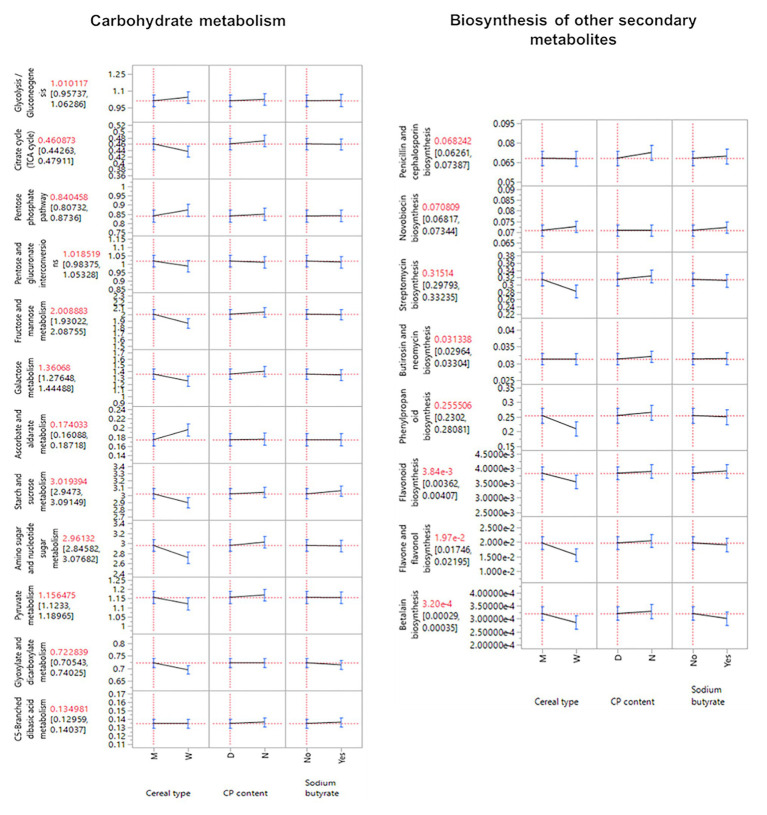
Predicted function for the categories carbohydrate metabolism and biosynthesis of other secondary metabolism. Only statistically significant features were included (*p* ≤ 0.05).

Functional predictions showed the strongest effect of the cereal type (Supplementary Table 7) and other statistical significances across all supplementations and their corresponding interactions (*p* ≤ 0.05; Supplementary Table 8). The influence of the cereal type was also observed in the microbial community composition (Supplementary Figure 1A). The pairwise comparison demonstrated that significant differences were present mainly when the cereal type changed. The broad categories of amino acid metabolism, carbohydrate metabolism, biosynthesis of other secondary metabolites, protein export, lipid metabolism, membrane transport were identified as the cause of the changes in predicted functions between the cereal types (Supplementary Table 8). Moreover, maize influenced a higher abundance of the predicted functions of the carbohydrate metabolism and biosynthesis of other secondary metabolites (*p* ≤ 0.05; Figure 5).

Some functions within amino acid metabolism differed significantly with sodium butyrate supplementation. Specifically, glycine, serine, and threonine metabolism had more abundance of genes in the absence of butyrate, while cysteine and methionine metabolism and lysine biosynthesis were higher in the presence of butyrate (Supplementary Table 8).

Crude protein content was significantly different for lysine degradation (amino acid metabolism) and galactose metabolism (Carbohydrate metabolism), where normal levels induced a higher abundance of genes (Supplementary Table 8).

## Discussion

Cereal type, CP levels, and presence/absence of sodium butyrate have a decisive impact on the gut microbial structure. Diet is one of the main contributors that influence both the host and its microbes (Borda-Molina et al., 2018; Makki et al., 2018). Therefore, different studies focused on the impact of cereal types on the gut microbiota (Maesschalck et al., 2019; Paraskeuas and Mountzouris, 2019), different levels of CP (Apajalahti and Vienola, 2016; Cesare et al., 2019) and different concentrations of sodium butyrate (Bortoluzzi et al., 2017; Wu et al., 2018). However, until now, there was no study testing the influence of these three essential dietary components in the same experimental trial.

In this work, the main contributor to differences in the community structure was the cereal supplementation. Wheat-based diets contain a high concentration of non-digestible polysaccharides, causing the need for supplementing enzymes like xylanase-glucanase mixtures to improve digestibility (Keyser et al., 2016). This could influence the activities and the composition of the microbial structure. *Bacteroides vulgatus* is a common colonizer of the chicken cecum (Torok et al., 2011), and it is known to metabolize starch (McCarthy et al., 1988). The lower dominance of *B. vulgatus* in wheat diets could be determined for possible effects on variations in apparent metabolizable energy (Crisol-Martínez et al., 2017).

*Bacteroides xylanisolvens* was promoted in the presence of WB diets. In the polysaccharides presented in cereals, there is a high content of xylans, which can be degraded to glucoronoarabinoxylans and arabinoxylans, through the xylanolytic activity exerted by *B. xylanisolvens* (Despres et al., 2016). Xylans and their derived compounds are considered to be prebiotic substrates that promote the presence of well-described beneficial bacteria *Lactobacillus* and *Bifidobacteria* (Despres et al., 2016). In this study, wheat promoted the presence of *Bifidobacterium pseudolongum*. *Bifidobacterium* spp. have been previously reported in higher levels in WB diets (Paraskeuas and Mountzouris, 2019). These species can use oligosaccharides from complex plant cell wall substrates due to the high glycosidase activity (van Laere et al., 2000). Lactobacillaceae is also known to develop fermentative activities with xylan and its compounds (Ratnadewi et al., 2020), which is in line with this study’s findings. Two of the most abundant OTUs (2 and 11) related to *Lactobacillus* species were also more abundant in WB diets.

Co-occurrence patterns investigated through network analysis are used to depict the microbes’ co-existence and maintenance in a determined environment (Williams et al., 2014). In this study, wheat promotes more neighborhood connectivity in comparison to MB diets. It is suggested that high interactions are promoted in more stable communities (Cardona et al., 2016). Therefore, it is interesting to observe that in wheat diets, *Lactobacillus, Bacteroides*, and unclassified Lachnospiraceae behave like keystone genera (Cardona et al., 2016) since they are detected in higher abundances and also have the more increased connectivity based on the microbial network analysis. Furthermore, *Lactobacillus* positively correlates with the birds’ body weight, and simultaneously high abundances were observed in those animals fed with WB diets.

Predicted functions determined cereal type as the most influencing factor. Biosynthesis of secondary metabolites and carbohydrate metabolites displayed more gene abundance for MB diets, which can be associated with the high nutritional value and the dietary preference in chickens for this cereal type (Kiarie et al., 2014). Maize has high concentrations of starch and lower contents of water-soluble NSP, antinutrient components that are much present in wheat (Ranjitkar et al., 2016). This could imply that more intense interactions need to be established to degrade complex substrates present in wheat, while more metabolic activities can be followed in easily degradable substrates present in maize cereals.

This study showed the influence of CP content on the microbial structure. Crude protein is essential in chicken metabolism since it constitutes the source of amino acids that are further absorbed and transformed into body proteins (Nakphaichit, 2014). Family Ruminococcaceae, recognized as a late colonizer of the chicken caeca (Richards et al., 2019), and family Lachnospiraceae were more abundant in the low level CP diets. Both families are major members of the phylum Firmicutes in the ceca; however, Ruminococcaceae is more abundant in birds with low feed conversion ratios (Singh et al., 2012) that can be associated in the present study to the lower levels of CP. The responses obtained by these family members confirm microbiota’s significant impact on feed digestion and assimilation of dietary components (Nakphaichit, 2014).

Butyrate is a source of energy to the intestinal epithelium, modulates the immune system, affects metabolism, and its depletion might cause the emergence of diseases by establishing enteric pathogens (Nicholson et al., 2012; Smith et al., 2013; Vital et al., 2017). Species of the family Bacteroidaceae have a large genome, which favors their adaptation to different environmental factors. The presence of carbohydrate-degrading enzymes allows the digestion of substrates from plant, algae, or animal sources. Together with the high tolerance to bile salts in the gut, these conditions influence a beneficial relationship with the host (Wexler, 2014). In this study, this family was affected by sodium butyrate and CP as an indicator of the microbes’ response to gut environmental conditions.

The present study showed the close interactions between microbial community composition, including the predicted functions and the complex feedstuff ingredients. It was observed that higher impacts were observed for maize as the main dietary cereal type promoting more abundant species. At the same time, wheat was associated with a higher abundance of well-recognized beneficial microorganisms belonging to Lactobacillaceae and Bifidobacteriaceae. Predicted functions demonstrated that maize could be considered the most potent cereal to promote the metabolism and biosynthesis of secondary metabolites. At the same time, genera in the WB diets have more interactions based on network connectivity due to the higher complexity of this cereal type.

## Data Availability Statement

The datasets presented in this study can be found in online repositories. The names of the repository/repositories and accession number(s) can be found at: https://www.ebi.ac.uk/ena, PRJEB40780.

## Ethics Statement

The animal study was reviewed and approved by Government Office of Pest County, Food Chain Safety, Plant Protection and Soil Conservation Directorate, Budapest, Hungary.

## Author Contributions

GM, ZN, KH, JS, and AC-S: conceptualization. DB-M, MM: methodology. DB-M, GM, and MM: performed the experiment. DB-M, JS, and AC-S: formal analysis. DB-M, GM, MM, ZN, KH, JS, and AC-S: writing—review and editing. ZN and AC-S: funding acquisition. All authors contributed to the article and approved the submitted version.

### Conflict of Interest

The authors declare that the research was conducted in the absence of any commercial or financial relationships that could be construed as a potential conflict of interest.
