# Supplementary material for: Caeca Microbial Variation in Broiler Chickens as a Result of Dietary Combinations Using Two Cereal Types, Supplementation of Crude Protein and Sodium Butyrate
Source: Front Microbiol. 2021 Jan 11;11:617800. doi: 10.3389/fmicb.2020.617800 (PMC7829199; doi:10.3389/fmicb.2020.617800)

Supplementary Material

1 Supplementary Figures and Tables

1.1 Supplementary Figures

Supplementary Figure 1. Cluster analysis based on the 8 dietary treatments. Colors indicate yellow for maize- based diets and blue for wheat based diets.

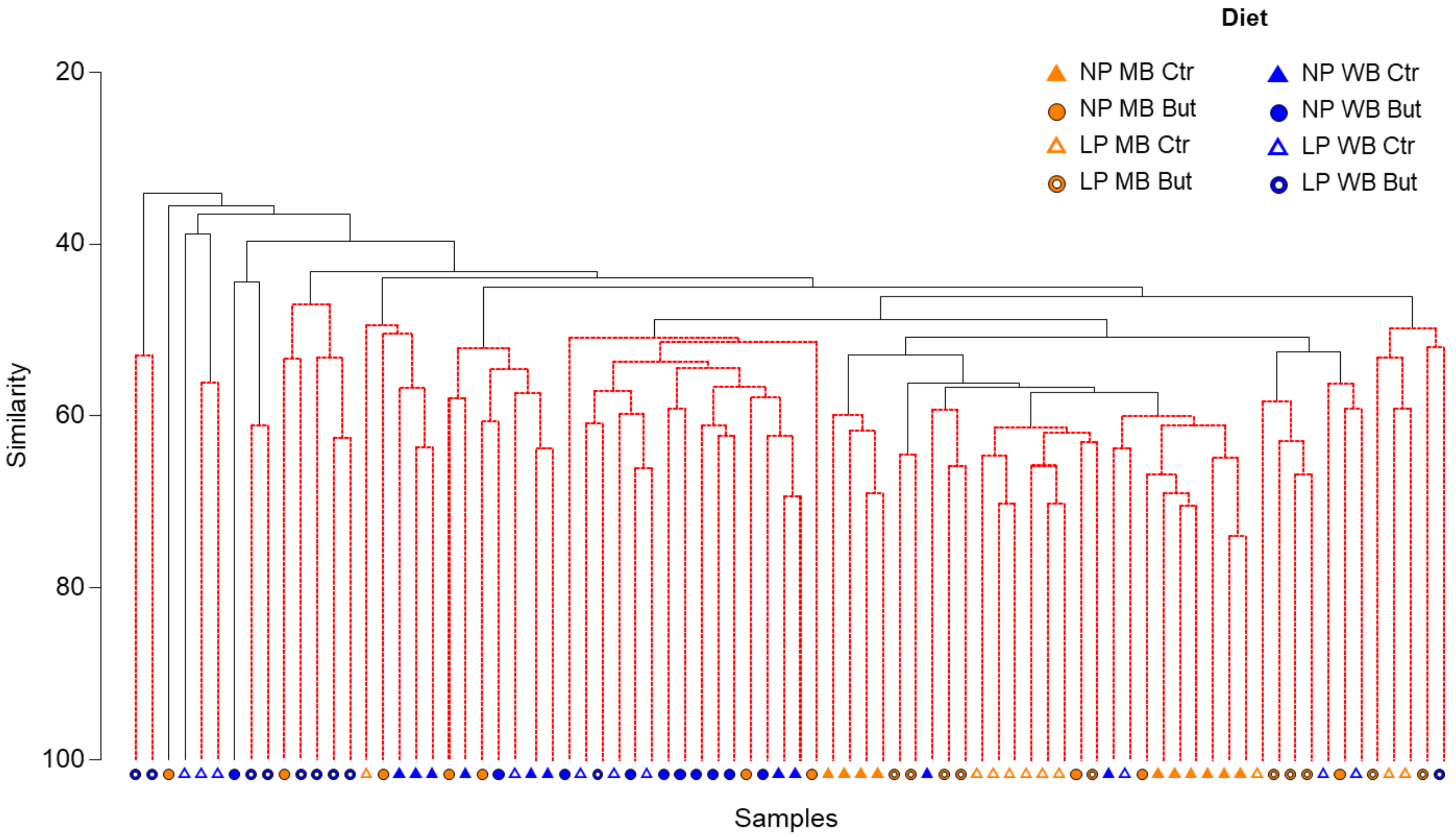

Supplementary Figure 2. Non-metrical dimensional scaling plot showing the microbial community distribution grouping the samples based on: A. Type of diet: maize (yellow) or wheat(blue); B. Crude protein content: normal (red) or low (green) and C. Sodium butyrate presence (gray) or absence (dark blue).

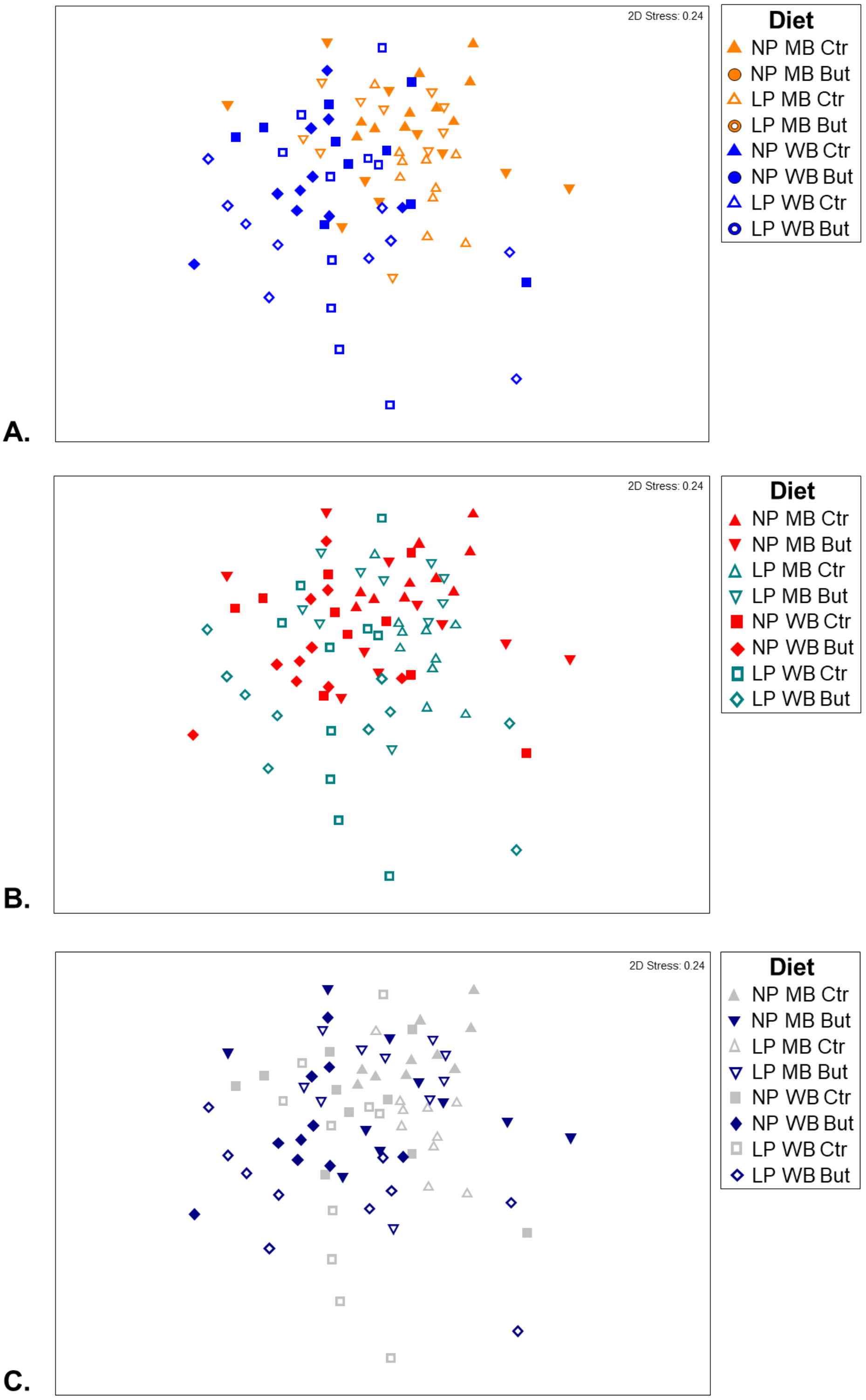

Supplementary Figure 3. Shannon diversity index for the eight dietary treatments.

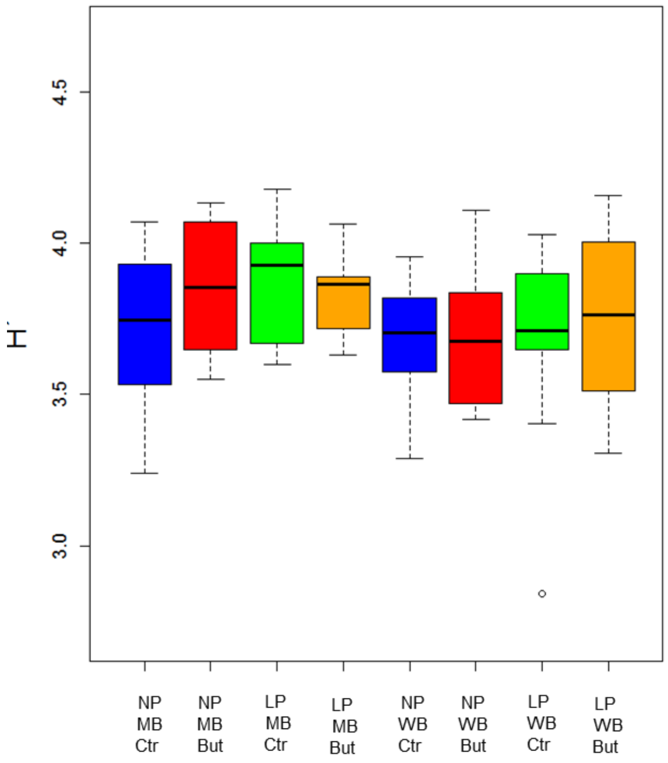

Supplementary Figure 4. Taxonomy classification for the eight dietary treatments at phylum level.

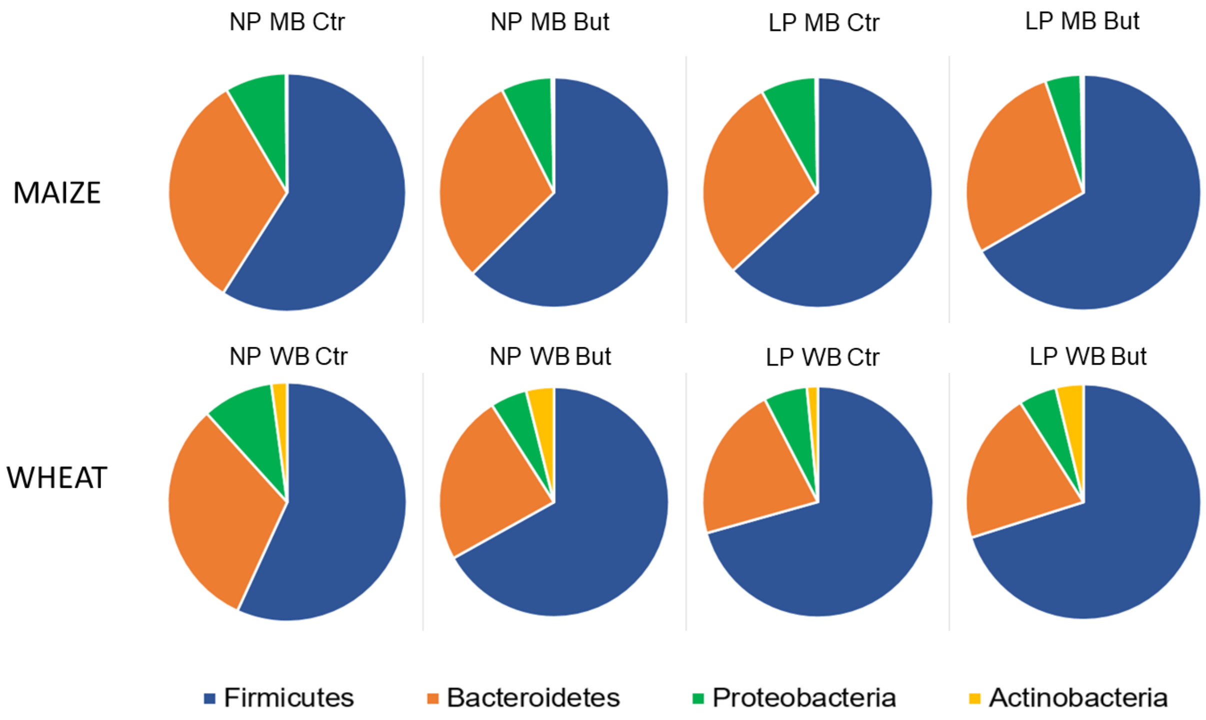

Supplementary Figure 5. Percentage of relative abundance for the eight dietary treatments at taxonomical levels A. Family and B. Genus.

A.

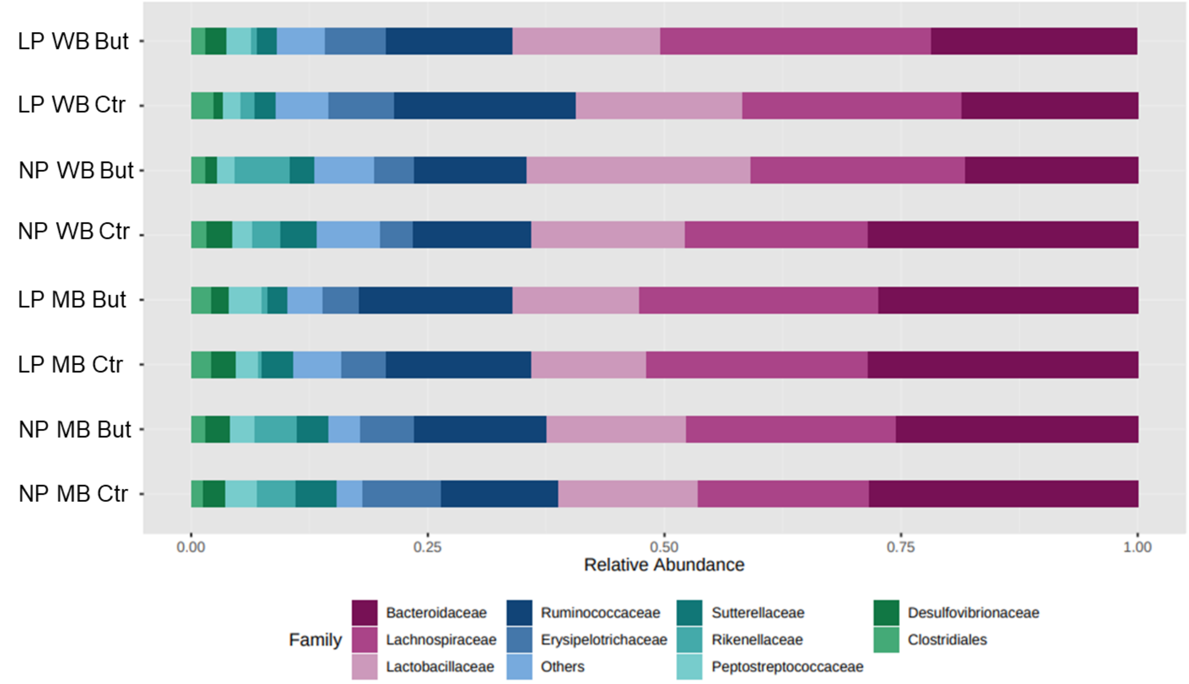

B.

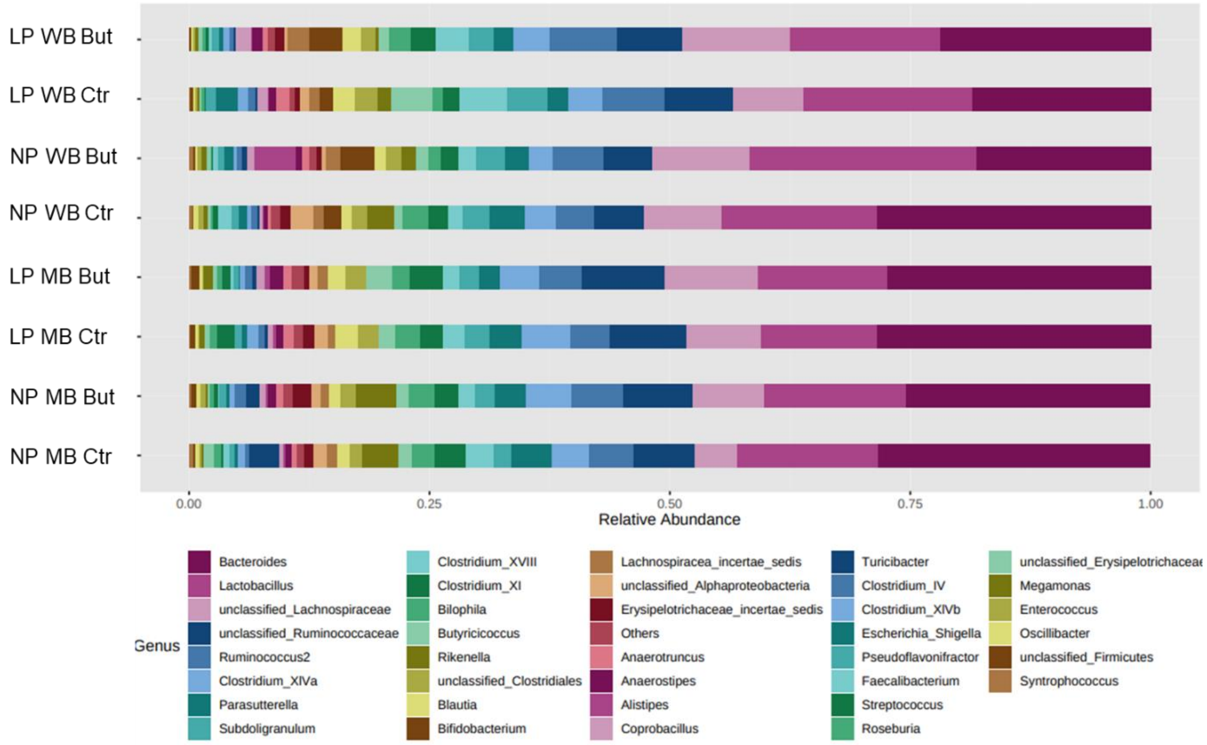

1.2 Supplementary Tables

Supplementary Table 1A. Composition of starter diet.

Ingredients		Maize-based				Wheat-based			
		Normal CP		Low CP		Normal CP		Low CP	
		Ctr	Butyrate	Ctr	Butyrate	Ctr	Butyrate	Ctr	Butyrate
Maize	%	57.6	57.6	61	61	0	0	0	0
Wheat	%	0	0	0	0	54.8	54.8	62.6	62.6
Extr, soybean meal	%	27	27	28	28	31	31	26.5	26.5
PL-68[1]	%	6.5	6.5	0	0	3	3	0	0
Sunflower oil	%	3.5	3.5	3.5	3.5	6	6	5.3	5.3
Wheat bran	%	0	0	1.72	1.72	0	0	0	0
Limestone	%	1.7	1.7	1.6	1.6	1.7	1.7	1.7	1.7
MCP	%	1.8	1.8	2	2	1.7	1.7	1.7	1.7
Salt (NaCl)	%	0.4	0.4	0.4	0.4	0.4	0.4	0.4	0.4
Lysine	%	0.44	0.44	0.58	0.58	0.38	0.38	0.6	0.6
Methionine	%	0.43	0.43	0.44	0.44	0.41	0.41	0.45	0.45
Threonine	%	0.09	0.09	0.22	0.22	0.11	0.11	0.26	0.26
Tryptophan	%	0.04	0.04	0.04	0.04	0	0	0	0
Vitamin and mineral premix[2]	%	0.5	0.5	0.5	0.5	0.5	0.5	0.5	0.5
Axtra XB 201 enzyme	%	0	0	0	0	0.015	0.015	0.015	0.015
Butyrate supplementation	%	0	0.15	0	0.15	0	0.15	0	0.15
Total		100	100	100	100	100	100	100	100

Supplementary Table 1B. Composition of grower diet.

Ingredients		Maize-based				Wheat-based			
		Normal CP		Low CP		Normal CP		Low CP	
		Ctr	Butyrate	Ctr	Butyrate	Ctr	Butyrate	Ctr	Butyrate
Maize	%	60.71	60.71	65.31	65.31	0	0	0	0
Wheat	%	0	0	0	0	61.3	61.3	66.56	66.56
Extr, soybean meal	%	22.2	22.2	24.54	24.54	19.31	19.31	20.01	20.01
PL-68[1]	%	8	8	1	1	8.5	8.5	2.5	2.5
Sunflower oil	%	4.8	4.8	4.5	4.5	6.7	6.7	6.5	6.5
Wheat bran	%	0	0	0	0	0	0	0	0
Limestone	%	1.3	1.3	1.2	1.2	1.35	1.35	1.35	1.35
MCP	%	1.35	1.35	1.6	1.6	1.15	1.15	1.15	1.15
Salt (NaCl)	%	0.4	0.4	0.4	0.4	0.4	0.4	0.4	0.4
Lysine	%	0.34	0.34	0.41	0.41	0.38	0.38	0.48	0.48
Methionine	%	0.36	0.36	0.37	0.37	0.35	0.35	0.38	0.38
Threonine	%	0	0	0.15	0.15	0.05	0.05	0.16	0.16
Tryptophan	%	0.04	0.04	0.02	0.02	0	0	0	0
Vitamin and mineral premix[2]	%	0.5	0.5	0.5	0.5	0.5	0.5	0.5	0.5
Axtra XB 201 enzyme	%	0	0	0	0	0.015	0.015	0.015	0.015
Butyrate supplementation	%	0	0.15	0	0.15	0	0.15	0	0.15
Total		100	100	100	100	100	100	100	100

Supplementary Table 2. Body weight data of broiler chickens (modified from Petrilla et al., 2018)

		Abbreviation of dietary group								
**Parameter**		**NP MB Ctr**	**NP MB But**	**LP MB Ctr**	**LP MB But**	**NP WB Ctr**	**NP WB But**	**LP WB Ctr**	**LP WB But**	**Significant differences**
**Body weight (g)**	Day 1	39.43 ±0.06	39.46 ±0.06	38.97 ±0.06	39.10 ±0.06	38.66 ±0.06	38.72 ±0.05	39.75 ±0.06	39.65 ±0.06	
	Day 7	171.0 ±8.4	179.8 ±4.9	174.9 ±7.1	179.6 ±8.1	177.6 ±9.4	184.7 ±8.4	196.6 ±9.9	211.4 ±9.9	**WB vs. MB , *LP vs. NP
	Day 21	679.5 ±20.8	638.1 ±34.1	824.3 ±32.9	864.0 ±28.6	826.0 ±37.2	845.6 ±30.7	821.2 ±34.0	811.6 ±33.7	**WB vs. MB, ***LP vs. NP
**Average daily body weight gain, day 1-7 (g/day)**		21.9	23.4	22.7	23.4	23.2	24.3	26.1	28.6	
**Average daily body weight**		36.3	32.7	46.4	48.9	46.3	47.2	44.6	42.9	

**gain, day 7-21 (g/day)**										
**Average daily body weight gain, day 21-42 (g/day)**		74.0	79.9	100.5	84.3	74.7	74.3	94.7	89.3	

Abbreviations of groups are presented in Supplementary Table 1. Results are expressed as mean ± SEM. Statistical analysis of data was performed by multi-way ANOVA test to evaluate main effects. Main effects were determined as follows: WB vs. MB diet, LP vs. NP groups and butyrate supplementation vs. no added butyrate.
***p<0.001; **p<0.01; *p<0.05

Supplementary Table 3. PERMANOVA results to inspect differences across microbial communities at the third week of dietary supplementation based on type of cereal (maize or wheat), the content of crude protein (normal or decreased) and sodium butyrate supplementation (with or without). Pairwise comparison was done based on t-test. Degrees of freedom (df), mean squares (MS), F values (F) and significance levels (P). Significant differences are highlighted in bold.

PERMANOVA table of results

Source	df	SS	MS	Pseudo-F	P(perm)
Cereal type	1	9270.2	9270.2	7.3892	**0.001**
Crude protein	1	5452.3	5452.3	4.3459	**0.001**
Sodium Butyrate	1	3189.1	3189.1	2.542	**0.003**
Ce x CP	1	2180.6	2180.6	1.7381	**0.026**
Ce x So	1	1559.7	1559.7	1.2432	0.179
CP x So	1	2284.3	2284.3	1.8208	**0.008**
Ce x CP x So	1	5053.1	5053.1	4.0278	**0.001**
Res	72	90329	1254.6		
Total	79	1.19E+05			

Pair-wise test

Groups	t	P(perm)
NP MB Ctr, Np MB But	1.8297	**0.001**
NP MB Ctr, LP MB Ctr	2.1884	**0.001**
NP MB Ctr, LP MB But	1.8589	**0.001**
NP MB Ctr, NP WB Ctr	2.2198	**0.001**

NP MB Ctr, NP WB But	2.4677	**0.001**
NP MB Ctr, LP WB Ctr	2.0617	**0.001**
NP MB Ctr, LP WB But	2.5517	**0.001**
NP MB But, LP MB Ctr	1.4803	**0.008**
NP MB But, LP MB But	1.5753	**0.002**
NP MB But, NP WB Ctr	1.3852	**0.027**
NP MB But, NP WB But	1.7913	**0.001**
NP MB But, LP WB Ctr	1.7279	**0.001**
NP MB But, LP WB But	1.6638	**0.002**
LP MB Ctr, LP MB But	1.4474	**0.005**
LP MB Ctr, NP WB Ctr	1.9437	**0.001**
LP MB Ctr, NP WB But	2.3434	**0.001**
LP MB Ctr, LP WB Ctr	1.7772	**0.001**
LP MB Ctr, LP WB But	1.8642	**0.001**
LP MB But, NP WB Ctr	1.9153	**0.001**
LP MB But, NP WB But	2.0781	**0.001**
LP MB But, LP WB Ctr	1.6321	**0.003**
LP MB But, LP WB But	1.8366	**0.001**
NP WB Ctr, NP WB But	1.5178	**0.004**
NP WB Ctr, LP WB Ctr	1.5831	**0.003**
NP WB Ctr, LP WB But	1.5604	**0.003**
NP WB But, LP WB Ctr	1.4193	**0.007**

NP WB But, LP WB But	1.6588	**0.001**
LP WB Ctr, LP WB But	1.4222	**0.005**

Average similarity within the pens

NP MB Ctr	NP MB But	LP MB Ctr	LP MB But	NP WB Ctr	NP WB But	LP WB Ctr	LP WB But
59%	47%	57%	53%	51%	52%	45%	43%

Supplementary Table 4. Univariate statistical analysis at family level based on ANOVA and multiple comparisons for the significant effects.

Summary effect of the three factors across all the families

Source	LogWorth	PValue
CP content	6.787	0.00000
Cereal type*CP content	4.111	0.00008
Cereal type	3.808	0.00016 ^
Cereal type*CP content*Sodium butyrate	2.805	0.00157
Sodium butyrate	2.554	0.00279 ^
CP content*Sodium butyrate	1.676	0.02107 ^
Cereal type*Sodium butyrate	1.600	0.02510 ^

Family Bacteroidaceae

Source	LogWorth	PValue
Cereal type	2.504	0.00313
CP content*Sodium butyrate	1.397	0.04013
Cereal type*CP content*Sodium butyrate	0.942	0.11428
Sodium butyrate	0.851	0.14103 ^

Source	LogWorth	PValue
Cereal type*CP content	0.562	0.27441 ^
CP content	0.238	0.57744 ^
Cereal type*Sodium butyrate	0.163	0.68727 ^

Term	Estimate	Std Error	t Ratio	Prob>|t|
Cereal type[M]	2.8288398	0.925245	3.06	0.0031*
CP content[D]	-0.517828	0.925245	-0.56	0.5774
Cereal type[M]*CP content[D]	1.0190247	0.925245	1.10	0.2744
Sodium butyrate[No]	1.3770605	0.925245	1.49	0.1410
Cereal type[M]*Sodium butyrate[No]	-0.373982	0.925245	-0.40	0.6873
CP content[D]*Sodium butyrate[No]	-1.934004	0.925245	-2.09	0.0401*
Cereal type[M]*CP content[D]*Sodium butyrate[No]	1.479131	0.925245	1.60	0.1143

CP content	Sodium butyrate	Estimate	Std Error	DF	Lower 95%	Upper 95%
D	No	23.536420	1.8504900	72	19.847536	27.225305
D	Yes	24.650308	1.8504900	72	20.961423	28.339192
N	No	28.440085	1.8504900	72	24.751201	32.128969
N	Yes	21.817956	1.8504900	72	18.129072	25.506840

Cereal type	Estimate	Std Error	DF	Lower 95%	Upper 95%
M	27.440032	1.3084940	72	24.831597	30.048467
W	21.782352	1.3084940	72	19.173917	24.390788

Family Bifidobacteriaceae

Source	LogWorth		PValue
Cereal type	3.808		0.00016
Sodium butyrate	0.863		0.13716
Cereal type*Sodium butyrate	0.828		0.14850
Cereal type*CP content	0.088		0.81622
CP content	0.087		0.81872 ^
CP content*Sodium butyrate	0.056		0.87940
Cereal type*CP content*Sodium butyrate	0.052		0.88798

Term	Estimate	Std Error	t Ratio	Prob>|t|
Cereal type[M]	-1.302369	0.32618	-3.99	0.0002*
CP content[D]	-0.075031	0.32618	-0.23	0.8187
Cereal type[M]*CP content[D]	0.0760855	0.32618	0.23	0.8162
Sodium butyrate[No]	-0.490317	0.32618	-1.50	0.1372
Cereal type[M]*Sodium butyrate[No]	0.4763906	0.32618	1.46	0.1485
CP content[D]*Sodium butyrate[No]	-0.049668	0.32618	-0.15	0.8794
Cereal type[M]*CP content[D]*Sodium butyrate[No]	0.0461109	0.32618	0.14	0.8880

Cereal type	Estimate	Std Error	DF	Lower 95%	Upper 95%
M	0.0184832	0.46128857	72	-0.901079	0.9380451
W	2.6232212	0.46128857	72	1.703659	3.5427832

Family Lachnospiraceae

Source	LogWorth	PValue
CP content	3.226	0.00059
Sodium butyrate	2.259	0.00550
Cereal type	0.443	0.36038
Cereal type*CP content*Sodium butyrate	0.406	0.39229
Cereal type*Sodium butyrate	0.235	0.58193 ^
Cereal type*CP content	0.111	0.77409 ^
CP content*Sodium butyrate	0.019	0.95659 ^

Term	Estimate	Std Error	t Ratio	Prob>|t|
Cereal type[M]	-0.57898	0.628973	-0.92	0.3604
CP content[D]	2.2597401	0.628973	3.59	0.0006*
Cereal type[M]*CP content[D]	-0.181212	0.628973	-0.29	0.7741
Sodium butyrate[No]	-1.800385	0.628973	-2.86	0.0055*
Cereal type[M]*Sodium butyrate[No]	0.3478607	0.628973	0.55	0.5819
CP content[D]*Sodium butyrate[No]	0.0343574	0.628973	0.05	0.9566

Term	Estimate	Std Error	t Ratio	Prob>|t|
Cereal type[M]*CP content[D]*Sodium butyrate[No]	0.541327	0.628973	0.86	0.3923

Sodium butyrate	Estimate	Std Error	DF	Lower 95%	Upper 95%
No	21.036986	0.88950222	72	19.263796	22.810177
Yes	24.637756	0.88950222	72	22.864566	26.410946

CP content	Estimate	Std Error	DF	Lower 95%	Upper 95%
D	25.097111	0.88950222	72	23.323921	26.870302
N	20.577631	0.88950222	72	18.804441	22.350822

Family Lactobacillaceae

Source	LogWorth	PValue
Cereal type	1.730	0.01863
Cereal type*CP content*Sodium butyrate	0.776	0.16760
CP content	0.766	0.17152 ^
CP content*Sodium butyrate	0.530	0.29545 ^
Sodium butyrate	0.442	0.36106 ^
Cereal type*Sodium butyrate	0.222	0.59970 ^

Source	LogWorth	PValue
Cereal type*CP content	0.148	0.71112 ^

Term	Estimate	Std Error	t Ratio	Prob>|t|
Cereal type[M]	-2.265357	0.94098	-2.41	0.0186*
CP content[D]	-1.299576	0.94098	-1.38	0.1715
Cereal type[M]*CP content[D]	0.3498781	0.94098	0.37	0.7111
Sodium butyrate[No]	-0.864946	0.94098	-0.92	0.3611
Cereal type[M]*Sodium butyrate[No]	0.4960525	0.94098	0.53	0.5997
CP content[D]*Sodium butyrate[No]	0.9917105	0.94098	1.05	0.2954
Cereal type[M]*CP content[D]*Sodium butyrate[No]	-1.311728	0.94098	-1.39	0.1676

Cereal type	Estimate	Std Error	DF	Lower 95%	Upper 95%
M	13.703728	1.3307470	72	11.050932	16.356523
W	18.234442	1.3307470	72	15.581646	20.887238

Family Rikenellaceae

Source	LogWorth	PValue
CP content	6.787	0.00000
CP content*Sodium butyrate	0.982	0.10435

Source	LogWorth	PValue
Cereal type*CP content*Sodium butyrate	0.891	0.12850
Sodium butyrate	0.533	0.29332 ^
Cereal type	0.244	0.56997 ^
Cereal type*Sodium butyrate	0.184	0.65489 ^
Cereal type*CP content	0.150	0.70794 ^

Term	Estimate	Std Error	t Ratio	Prob>|t|
Cereal type[M]	-0.176461	0.309194	-0.57	0.5700
CP content[D]	-1.793601	0.309194	-5.80	<.0001*
Cereal type[M]*CP content[D]	-0.116291	0.309194	-0.38	0.7079
Sodium butyrate[No]	-0.327314	0.309194	-1.06	0.2933
Cereal type[M]*Sodium butyrate[No]	0.1387795	0.309194	0.45	0.6549
CP content[D]*Sodium butyrate[No]	0.5085932	0.309194	1.64	0.1043
Cereal type[M]*CP content[D]*Sodium butyrate[No]	-0.475453	0.309194	-1.54	0.1285

CP content	Estimate	Std Error	DF	Lower 95%	Upper 95%
D	0.7638797	0.43726568	72	-0.107793	1.6355529
N	4.3510815	0.43726568	72	3.479408	5.2227547

Family Ruminococcaceae

Source	LogWorth		PValue
CP content	1.860		0.01380
Cereal type*Sodium butyrate	1.017		0.09624
CP content*Sodium butyrate	0.647		0.22567
Cereal type*CP content*Sodium butyrate	0.445		0.35889
Sodium butyrate	0.349		0.44789 ^
Cereal type*CP content	0.224		0.59696 ^
Cereal type	0.040		0.91243 ^

Term	Estimate	Std Error	t Ratio	Prob>|t|
Cereal type[M]	0.0705548	0.639324	0.11	0.9124
CP content[D]	1.6138129	0.639324	2.52	0.0138*
Cereal type[M]*CP content[D]	-0.339566	0.639324	-0.53	0.5970
Sodium butyrate[No]	0.4878762	0.639324	0.76	0.4479
Cereal type[M]*Sodium butyrate[No]	-1.077522	0.639324	-1.69	0.0962
CP content[D]*Sodium butyrate[No]	0.781286	0.639324	1.22	0.2257
Cereal type[M]*CP content[D]*Sodium butyrate[No]	-0.590349	0.639324	-0.92	0.3589

CP content	Estimate	Std Error	DF	Lower 95%	Upper 95%
D	16.015683	0.90414105	72	14.213311	17.818055
N	12.788057	0.90414105	72	10.985685	14.590429

Supplementary Table 5. Univariate statistical analysis at OTU level based on ANOVA and multiple comparisons for the significant effects.

Summary effects of the three factors across all the OTUs

Source	LogWorth	PValue
Diet type	9.000	0.00000
CP content	6.607	0.00000
Diet type*Sodium butyrate	5.239	0.00001
Diet type*CP content*Sodium butyrate	5.041	0.00001
CP content*Sodium butyrate	4.623	0.00002 ^
Sodium butyrate	4.505	0.00003 ^
Diet type*CP content	3.851	0.00014 ^

OTU1 – *Bacteroides vulgatus*

Source	LogWorth	PValue
Cereal type	4.100	0.00008
Cereal type*CP content*Sodium butyrate	2.190	0.00645
Sodium butyrate	2.109	0.00778 ^
CP content*Sodium butyrate	1.014	0.09692 ^
Cereal type*CP content	0.251	0.56076 ^
CP content	0.175	0.66838 ^

Source	LogWorth	PValue
Cereal type*Sodium butyrate	0.141	0.72319 ^

Term	Estimate	Std Error	t Ratio	Prob>|t|
Cereal type[M]	3.1396709	0.750195	4.19	<.0001*
CP content[D]	-0.322685	0.750195	-0.43	0.6684
Cereal type[M]*CP content[D]	0.4384301	0.750195	0.58	0.5608
Sodium butyrate[No]	2.0542756	0.750195	2.74	0.0078*
Cereal type[M]*Sodium butyrate[No]	0.266754	0.750195	0.36	0.7232
CP content[D]*Sodium butyrate[No]	-1.261771	0.750195	-1.68	0.0969
Cereal type[M]*CP content[D]*Sodium butyrate[No]	-2.104722	0.750195	-2.81	0.0065*

Cereal type	CP content	Sodium butyrate	Estimate	Std Error	DF	Lower 95%	Upper 95%
M	D	No	16.499633	2.1218705	72	12.269762	20.729505
M	D	Yes	18.590561	2.1218705	72	14.360689	22.820432
M	N	No	23.001130	2.1218705	72	18.771258	27.231002
M	N	Yes	11.626084	2.1218705	72	7.396213	15.855956
W	D	No	13.019367	2.1218705	72	8.789496	17.249239
W	D	Yes	7.758422	2.1218705	72	3.528551	11.988294
W	N	No	12.855696	2.1218705	72	8.625825	17.085568
W	N	Yes	10.966555	2.1218705	72	6.736683	15.196426

OTU2 – *Lactobacillus crispatus*

Source	LogWorth	PValue
Cereal type	3.219	0.00060
CP content*Sodium butyrate	1.536	0.02908
CP content	1.308	0.04926 ^
Cereal type*CP content	1.169	0.06774
Cereal type*CP content*Sodium butyrate	0.670	0.21370
Cereal type*Sodium butyrate	0.111	0.77520 ^
Sodium butyrate	0.045	0.90145 ^

Term	Estimate	Std Error	t Ratio	Prob>|t|
Cereal type[M]	-1.735116	0.483533	-3.59	0.0006*
CP content[D]	-0.967133	0.483533	-2.00	0.0493*
Cereal type[M]*CP content[D]	0.8967744	0.483533	1.85	0.0677
Sodium butyrate[No]	-0.06009	0.483533	-0.12	0.9014
Cereal type[M]*Sodium butyrate[No]	0.1386087	0.483533	0.29	0.7752
CP content[D]*Sodium butyrate[No]	1.0767758	0.483533	2.23	0.0291*
Cereal type[M]*CP content[D]*Sodium butyrate[No]	-0.606615	0.483533	-1.25	0.2137

CP content	Sodium butyrate	Estimate	Std Error	DF	Lower 95%	Upper 95%
D	No	8.112832	0.96706590	72	6.1850216	10.040643
D	Yes	6.079460	0.96706590	72	4.1516495	8.007271
N	No	7.893547	0.96706590	72	5.9657361	9.821357
N	Yes	10.167278	0.96706590	72	8.2394674	12.095089

OTU3 – *Bacteroides xylanisolvens*

Source	LogWorth	PValue	
Cereal type*CP content*Sodium butyrate	3.117	0.00076	
CP content	0.870	0.13505	^
Cereal type*Sodium butyrate	0.380	0.41657	^
Sodium butyrate	0.354	0.44236	^
Cereal type*CP content	0.086	0.81952	^
CP content*Sodium butyrate	0.073	0.84545	^
Cereal type	0.045	0.90114	^

Term	Estimate	Std Error	t Ratio	Prob>|t|
Cereal type[M]	0.0871222	0.698847	0.12	0.9011
CP content[D]	1.0562719	0.698847	1.51	0.1351
Cereal type[M]*CP content[D]	-0.160031	0.698847	-0.23	0.8195
Sodium butyrate[No]	-0.53984	0.698847	-0.77	0.4424

Term	Estimate	Std Error	t Ratio	Prob>|t|
Cereal type[M]*Sodium butyrate[No]	0.571022	0.698847	0.82	0.4166
CP content[D]*Sodium butyrate[No]	-0.136718	0.698847	-0.20	0.8454
Cereal type[M]*CP content[D]*Sodium butyrate[No]	2.4565418	0.698847	3.52	0.0008*

Cereal type	CP content	Sodium butyrate	Estimate	Std Error	DF	Lower 95%	Upper 95%
M	D	No	9.813145	1.9766374	72	5.872791	13.753500
M	D	Yes	5.111135	1.9766374	72	1.170780	9.051489
M	N	No	3.381016	1.9766374	72	-0.559338	7.321371
M	N	Yes	7.958299	1.9766374	72	4.017944	11.898653
W	D	No	3.903835	1.9766374	72	-0.036520	7.844189
W	D	Yes	11.312079	1.9766374	72	7.371725	15.252434
W	N	No	6.657750	1.9766374	72	2.717395	10.598105
W	N	Yes	3.692954	1.9766374	72	-0.247401	7.633308

OTU4 – *Lactobacillus salivarius*

Source	LogWorth	PValue
Cereal type*CP content*Sodium butyrate	1.951	0.01119
Cereal type*CP content	0.749	0.17829 ^
CP content	0.536	0.29115 ^
Sodium butyrate	0.175	0.66788 ^

Source	LogWorth	PValue
Cereal type	0.064	0.86333 ^
Cereal type*Sodium butyrate	0.048	0.89562 ^
CP content*Sodium butyrate	0.009	0.97938 ^

Term	Estimate	Std Error	t Ratio	Prob>|t|
Cereal type[M]	0.0706323	0.40886	0.17	0.8633
CP content[D]	-0.434783	0.40886	-1.06	0.2912
Cereal type[M]*CP content[D]	-0.555762	0.40886	-1.36	0.1783
Sodium butyrate[No]	-0.176146	0.40886	-0.43	0.6679
Cereal type[M]*Sodium butyrate[No]	0.0538323	0.40886	0.13	0.8956
CP content[D]*Sodium butyrate[No]	0.0106027	0.40886	0.03	0.9794
Cereal type[M]*CP content[D]*Sodium butyrate[No]	-1.064635	0.40886	-2.60	0.0112*

Cereal type	CP content	Sodium butyrate	Estimate	Std Error	DF	Lower 95%	Upper 95%
M	D	No	2.4762573	1.1564303	72	0.1709557	4.7815590
M	D	Yes	4.8289486	1.1564303	72	2.5236470	7.1342503
M	N	No	6.5654121	1.1564303	72	4.2601104	8.8707137
M	N	Yes	4.7019758	1.1564303	72	2.3966742	7.0072775
W	D	No	5.4681214	1.1564303	72	3.1628197	7.7734230

Cereal type	CP content	Sodium butyrate	Estimate	Std Error	DF	Lower 95%	Upper 95%
W	D	Yes	3.7776036	1.1564303	72	1.4723019	6.0829052
W	N	No	3.0756896	1.1564303	72	0.7703880	5.3809913
W	N	Yes	5.6861212	1.1564303	72	3.3808196	7.9914229

OTU6 – Uncultured *Parasuterella*

Source	LogWorth	PValue
CP content	2.447	0.00357
Sodium butyrate	2.119	0.00760
Cereal type	0.676	0.21067
Cereal type*CP content*Sodium butyrate	0.580	0.26324
Cereal type*Sodium butyrate	0.278	0.52774 ^
CP content*Sodium butyrate	0.098	0.79853 ^
Cereal type*CP content	0.053	0.88486 ^

Term	Estimate	Std Error	t Ratio	Prob>|t|
Cereal type[M]	0.0706323	0.40886	0.17	0.8633
CP content[D]	-0.434783	0.40886	-1.06	0.2912
Cereal type[M]*CP content[D]	-0.555762	0.40886	-1.36	0.1783
Sodium butyrate[No]	-0.176146	0.40886	-0.43	0.6679

Term	Estimate	Std Error	t Ratio	Prob>|t|
Cereal type[M]*Sodium butyrate[No]	0.0538323	0.40886	0.13	0.8956
CP content[D]*Sodium butyrate[No]	0.0106027	0.40886	0.03	0.9794
Cereal type[M]*CP content[D]*Sodium butyrate[No]	-1.064635	0.40886	-2.60	0.0112*

Cereal type	CP content	Sodium butyrate	Estimate	Std Error	DF	Lower 95%	Upper 95%
M	D	No	2.4762573	1.1564303	72	0.1709557	4.7815590
M	D	Yes	4.8289486	1.1564303	72	2.5236470	7.1342503
M	N	No	6.5654121	1.1564303	72	4.2601104	8.8707137
M	N	Yes	4.7019758	1.1564303	72	2.3966742	7.0072775
W	D	No	5.4681214	1.1564303	72	3.1628197	7.7734230
W	D	Yes	3.7776036	1.1564303	72	1.4723019	6.0829052
W	N	No	3.0756896	1.1564303	72	0.7703880	5.3809913
W	N	Yes	5.6861212	1.1564303	72	3.3808196	7.9914229

OTU11 – *Lactobacillus vaginalis*

Source	LogWorth	PValue
Cereal type	1.316	0.04833
Cereal type*CP content*Sodium butyrate	0.954	0.11129
Sodium butyrate	0.933	0.11662 ^

Source	LogWorth		PValue
CP content*Sodium butyrate	0.588		0.25815 ^
Cereal type*CP content	0.213		0.61294 ^
Cereal type*Sodium butyrate	0.192		0.64318 ^
CP content	0.060		0.87038 ^

Term	Estimate	Std Error	t Ratio	Prob>|t|
Cereal type[M]	-0.512162	0.254987	-2.01	0.0483*
CP content[D]	0.0417565	0.254987	0.16	0.8704
Cereal type[M]*CP content[D]	-0.129559	0.254987	-0.51	0.6129
Sodium butyrate[No]	-0.404979	0.254987	-1.59	0.1166
Cereal type[M]*Sodium butyrate[No]	0.1186255	0.254987	0.47	0.6432
CP content[D]*Sodium butyrate[No]	-0.290633	0.254987	-1.14	0.2581
Cereal type[M]*CP content[D]*Sodium butyrate[No]	0.4110919	0.254987	1.61	0.1113

Cereal type	Estimate	Std Error	DF	Lower 95%	Upper 95%
M	1.4138395	0.36060675	72	0.6949831	2.1326959
W	2.4381644	0.36060675	72	1.7193080	3.1570208

OTU22 – *Bifidobacterium pseudolongum*

Source	LogWorth	PValue
Cereal type	3.788	0.00016
Sodium butyrate	0.854	0.14002
Cereal type*Sodium butyrate	0.820	0.15137
Cereal type*CP content	0.084	0.82396
CP content	0.083	0.82568 ^
CP content*Sodium butyrate	0.059	0.87222
Cereal type*CP content*Sodium butyrate	0.055	0.88031

Term	Estimate	Std Error	t Ratio	Prob>|t|
Cereal type[M]	-1.299402	0.326502	-3.98	0.0002*
CP content[D]	-0.072171	0.326502	-0.22	0.8257
Cereal type[M]*CP content[D]	0.072899	0.326502	0.22	0.8240
Sodium butyrate[No]	-0.487198	0.326502	-1.49	0.1400
Cereal type[M]*Sodium butyrate[No]	0.4734642	0.326502	1.45	0.1514
CP content[D]*Sodium butyrate[No]	-0.052701	0.326502	-0.16	0.8722
Cereal type[M]*CP content[D]*Sodium butyrate[No]	0.0493377	0.326502	0.15	0.8803

Cereal type	Estimate	Std Error	DF	Lower 95%	Upper 95%
M	0.0181560	0.46174409	72	-0.902314	0.9386260
W	2.6169608	0.46174409	72	1.696491	3.5374309

Supplementary Table 6. Correlations between the most relevant OTUs and performance parameters (Body weight [g] – caecum length [cm]) for the eight dietary treatments.

OTU	OTU	*p* value	Pearson correlation
Bacteroides vulgatus OTU1	*Bacteroides xylanisolvens* - OTU3	0.030	-0.25
	uncultured *Parasutterella* - OTU6	0.013	0.27
	uncultured *Ruminococcus* - OTU8	0.004	-0.31
	unclassified Lachnospiraceae - OTU9	0.001	-0.34
	uncultured *Fusicatenibacter* - OTU13	0.024	-0.25
	unclassified *Clostridium* XIVa - OTU17	0.042	-0.22
	uncultured *Bacteroides* - OTU20	0.017	-0.26
	Bifidobacterium pseudolongum - OTU22	0.023	-0.25
	uncultured *Rhodospirillaceae* - OTU26	0.022	0.25
	unclassified *Bacteroides* - OTU28	0.014	-0.27
	uncultured *Coprobacillus* - OTU29	0.008	-0.3
	uncultured *Turicibacter* - OTU30	0.004	0.31
	uncultured *Fusicatenibacter* - OTU31	0.017	-0.26
	uncultured *Clostridium* XVIII - OTU32	0.035	-0.23
	Unc. Lachnospiracea incertae sedis - OTU33	0.011	-0.28
	uncultured *Clostridium* XlVa - OTU52	0.019	0.26

	uncultured *Ruminococcus*2 – OTU54	0.045	-0.22
	Caecum length (cm.)	0.050	-0.30
Lactobacillus crispatus OTU2	*Lactobacillus salivarius* - OTU4	0.0005	0.38
	uncultured *Bilophilla* - OTU10	0.0001	-0.42
	Lactobacillus vaginalis - OTU11	< 0.01	0.43
	uncultured *Clostridium* IV - OTU18	0.012	-0.28
	uncultured *Rikenella* - OTU19	0.016	-0.27
	uncultured *Lactobacillus* - OTU21	0.02	0.26
	uncultured *Turicibacter* - OTU30	0.012	-0.28
	uncultured *Rikenella* - OTU39	0.009	-0.29
	Unclassified *Lachnospiraceae* – OTU44	0.001	-0.28
	uncultured Pseudoflavonifractor - OTU46	0.020	-0.26
	uncultured Desulfotomaculum - OTU47	0.037	-0.23
	uncultured Allistipes - OTU49	0.043	0.22
	Unclassified *Lactobacillus* - OTU51	< 0.01	0.53
	unclassified Clostridium XIVa - OTU 52	0.001	-0.36
	Body weight (g.)	0.044	0.22
Uncultured *Ruminococcus* OTU8	*Bacteroides vulgatus* - OTU1	0.005	-0.31
	uncultured *Allistipes* - OTU5	< 0.01	0.52
	uncultured *Fusicatenibacter* - OTU13	0.001	0.36

	unclassified *Barnesiella* - OTU16	< 0.01	0.51
	uncultured *Bacteroides* - OTU28	0.003	0.33
	uncultured *Coprobacillus* - OTU29	0.0002	0.41
	uncultured *Fusicatenibacter* - OTU31	0.003	0.32
	Unc. Lachnospiracea incertae sedis - OTU33	5.00E-05	0.44
	uncultured *Anaerostipes* - OTU35	0.0002	0.41
	unclassified *Clostridium* XIVa - OTU42	0.002	0.34
	uncultured *Ruminococcus*2 – OTU54	< 0.01	0.65
	Caecum length (cm.)	0.001	0.34
Lactobacillus crispatus OTU11	Body weight (g)	0.01	0.26
	Caecum length (cm.)	< 0.01	0.36

Supplementary Table 7. PERMANOVA results for the functional prediction of the microbial communities at the third week of dietary supplementation based on type of cereal (maize or wheat), the content of crude protein (normal or decreased) and sodium butyrate supplementation (with or without). Pairwise comparison was done based on t-test. Degrees of freedom (df), mean squares (MS), F values (F) and significance levels (P). Not significant differences are highlighted in bold.

PERMANOVA table of results

Source	df	SS	MS	Pseudo-F	P(perm)
Cereal type	1	76.715	76.715	7.6702	**0.0003**
Crude protein	1	20.503	20.503	2.0499	0.1091
Sodium Butyrate	1	21.167	21.167	2.1164	0.0991
Ce x CP	1	8.9255	8.9255	0.8924	0.4219
Ce x So	1	9.3215	9.3215	0.93199	0.4068
CP x So	1	17.019	17.019	1.7016	0.1645
Ce x CP x So	1	8.7723	8.7723	0.87708	0.4328
Res	72	720.12	10.002		
Total	79	882.55			

Pair-wise test

Groups	t	P(perm)
NP MB Ctr, NP WB But	1.784	**0.039**

NP MB Ctr, LP WB Ctr	2.090	**0.012**
NP WB Ctr, LP WB Ctr	2.056	**0.010**
NP MB But, NP WB But	1.798	**0.037**
NP MB But, LP WB Ctr	1.859	**0.035**
LP MB Ctr, NP WB But	2.435	**0.002**
LP MB Ctr, LP WB Ctr	2.038	**0.002**
LP MB But, NP WB But	1.993	**0.022**
LP MB But, LP WB Ctr	2.057	**0.012**
NP WB But, LP WB Ctr	1.689	**0.042**

Supplementary Table 8. Significance for the most relevant predicted functions and source of the most influencing factor.

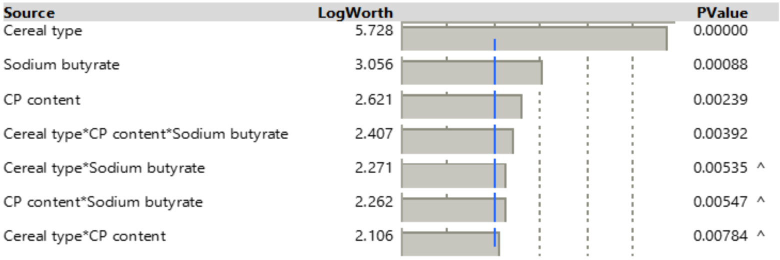

Source	LogWorth	PValue
Cereal type	5.728	0.00000
Sodium butyrate	3.056	0.00088
CP content	2.621	0.00239
Cereal type*CP content*Sodium butyrate	2.407	0.00392
Cereal type*Sodium butyrate	2.271	0.00535 ^
CP content*Sodium butyrate	2.262	0.00547 ^
Cereal type*CP content	2.106	0.00784 ^

Amino acid metabolism

Glycine serine threonine metabolism

Term	Estimate	Std Error	t Ratio	Prob>|t|
Cereal type[M]	-0.003048	0.002533	-1.20	0.2327
CP content[D]	-0.002964	0.002533	-1.17	0.2459
Cereal type[M]*CP content[D]	-0.001317	0.002533	-0.52	0.6047
Sodium butyrate[No]	0.0061643	0.002533	2.43	0.0174*
Cereal type[M]*Sodium butyrate[No]	-0.003086	0.002533	-1.22	0.2271
CP content[D]*Sodium butyrate[No]	0.0027415	0.002533	1.08	0.2827
Cereal type[M]*CP content[D]*Sodium butyrate[No]	-0.002304	0.002533	-0.91	0.3662

Cysteine and methionine metabolism

Term	Estimate	Std Error	t Ratio	Prob>|t|
Cereal type[M]	-0.008166	0.005221	-1.56	0.1222
CP content[D]	-0.003073	0.005221	-0.59	0.5580

Term	Estimate	Std Error	t Ratio	Prob>|t|
Cereal type[M]*CP content[D]	0.001298	0.005221	0.25	0.8044
Sodium butyrate[No]	-0.013931	0.005221	-2.67	0.0094*
Cereal type[M]*Sodium butyrate[No]	0.0072971	0.005221	1.40	0.1665
CP content[D]*Sodium butyrate[No]	-0.005873	0.005221	-1.12	0.2644
Cereal type[M]*CP content[D]*Sodium butyrate[No]	0.0029691	0.005221	0.57	0.5713

Valine, leucine isoleucine degradation

Term	Estimate	Std Error	t Ratio	Prob>|t|
Cereal type[M]	0.0077281	0.003326	2.32	0.0230*
CP content[D]	-0.005439	0.003326	-1.64	0.1063
Cereal type[M]*CP content[D]	0.0001501	0.003326	0.05	0.9641
Sodium butyrate[No]	0.0019099	0.003326	0.57	0.5676
Cereal type[M]*Sodium butyrate[No]	-0.000988	0.003326	-0.30	0.7672
CP content[D]*Sodium butyrate[No]	-0.002863	0.003326	-0.86	0.3922
Cereal type[M]*CP content[D]*Sodium butyrate[No]	0.0016202	0.003326	0.49	0.6276

Valine, leucine and isoleucine biosynthesis

Term	Estimate	Std Error	t Ratio	Prob>|t|
Cereal type[M]	-0.009663	0.003115	-3.10	0.0027*
CP content[D]	0.0051886	0.003115	1.67	0.1001

Term	Estimate	Std Error	t Ratio	Prob>|t|
Cereal type[M]*CP content[D]	-0.000337	0.003115	-0.11	0.9141
Sodium butyrate[No]	0.0001241	0.003115	0.04	0.9683
Cereal type[M]*Sodium butyrate[No]	-0.000873	0.003115	-0.28	0.7802
CP content[D]*Sodium butyrate[No]	0.0025328	0.003115	0.81	0.4189
Cereal type[M]*CP content[D]*Sodium butyrate[No]	-0.002115	0.003115	-0.68	0.4993

Lysine biosynthesis

Term	Estimate	Std Error	t Ratio	Prob>|t|
Cereal type[M]	-0.013884	0.003383	-4.10	0.0001*
CP content[D]	-0.002373	0.003383	-0.70	0.4852
Cereal type[M]*CP content[D]	0.0026044	0.003383	0.77	0.4438
Sodium butyrate[No]	-0.00754	0.003383	-2.23	0.0289*
Cereal type[M]*Sodium butyrate[No]	0.0046536	0.003383	1.38	0.1732
CP content[D]*Sodium butyrate[No]	-0.000709	0.003383	-0.21	0.8346
Cereal type[M]*CP content[D]*Sodium butyrate[No]	-0.000382	0.003383	-0.11	0.9104

Lysine degradation

Term	Estimate	Std Error	t Ratio	Prob>|t|
Cereal type[M]	0.0068444	0.003979	1.72	0.0897
CP content[D]	-0.008095	0.003979	-2.03	0.0456*

Term	Estimate	Std Error	t Ratio	Prob>|t|
Cereal type[M]*CP content[D]	0.0045228	0.003979	1.14	0.2594
Sodium butyrate[No]	0.0008886	0.003979	0.22	0.8239
Cereal type[M]*Sodium butyrate[No]	0.0018931	0.003979	0.48	0.6357
CP content[D]*Sodium butyrate[No]	-0.00138	0.003979	-0.35	0.7297
Cereal type[M]*CP content[D]*Sodium butyrate[No]	-0.00124	0.003979	-0.31	0.7562

Tyrosine metabolism

Term	Estimate	Std Error	t Ratio	Prob>|t|
Cereal type[M]	-0.008748	0.003037	-2.88	0.0052*
CP content[D]	0.0048858	0.003037	1.61	0.1121
Cereal type[M]*CP content[D]	-0.003412	0.003037	-1.12	0.2650
Sodium butyrate[No]	0.002497	0.003037	0.82	0.4137
Cereal type[M]*Sodium butyrate[No]	-0.004487	0.003037	-1.48	0.1440
CP content[D]*Sodium butyrate[No]	0.0084887	0.003037	2.79	0.0067*
Cereal type[M]*CP content[D]*Sodium butyrate[No]	-0.005795	0.003037	-1.91	0.0604

Phenylalanine metabolism

Term	Estimate	Std Error	t Ratio	Prob>|t|
Cereal type[M]	0.0049308	0.002266	2.18	0.0328*
CP content[D]	-0.000156	0.002266	-0.07	0.9452

Term	Estimate	Std Error	t Ratio	Prob>|t|
Cereal type[M]*CP content[D]	-0.00208	0.002266	-0.92	0.3617
Sodium butyrate[No]	-0.001036	0.002266	-0.46	0.6490
Cereal type[M]*Sodium butyrate[No]	-0.002457	0.002266	-1.08	0.2819
CP content[D]*Sodium butyrate[No]	-0.001339	0.002266	-0.59	0.5565
Cereal type[M]*CP content[D]*Sodium butyrate[No]	0.0019007	0.002266	0.84	0.4044

Phenylalanine, tyrosine and tryptophan biosynthesis

Term	Estimate	Std Error	t Ratio	Prob>|t|
Cereal type[M]	-0.012541	0.005528	-2.27	0.0263*
CP content[D]	0.0034922	0.005528	0.63	0.5296
Cereal type[M]*CP content[D]	0.0004516	0.005528	0.08	0.9351
Sodium butyrate[No]	-0.010513	0.005528	-1.90	0.0612
Cereal type[M]*Sodium butyrate[No]	0.0054415	0.005528	0.98	0.3282
CP content[D]*Sodium butyrate[No]	-0.004593	0.005528	-0.83	0.4088
Cereal type[M]*CP content[D]*Sodium butyrate[No]	0.0029413	0.005528	0.53	0.5963

Source	LogWorth		PValue
Cereal type	3.977	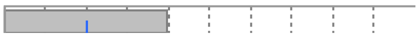	0.00011

Source	LogWorth	PValue
CP content*Sodium butyrate	2.177	0.00665
Sodium butyrate	2.026	0.00941 ^
CP content	1.341	0.04558 ^
Cereal type*CP content*Sodium butyrate	1.219	0.06039
Cereal type*Sodium butyrate	0.842	0.14397 ^
Cereal type*CP content	0.586	0.25942 ^

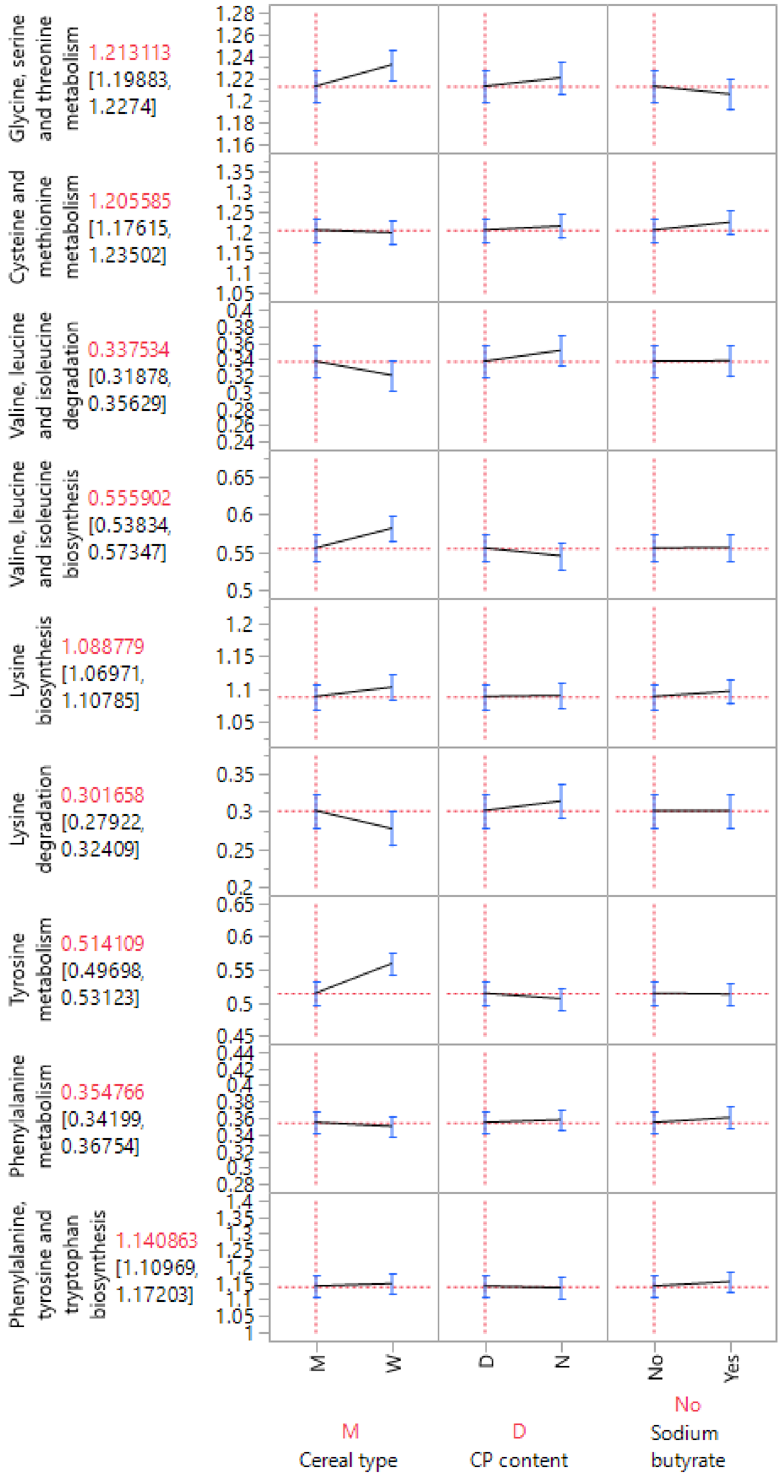
Glycine, serine and threonine metabolism
1.213113
[1.19883, 1.2274]
Cysteine and methionine metabolism
1.205585
[1.17615, 1.23502]
Valine, leucine and isoleucine degradation
0.337534
[0.31878, 0.35629]
Valine, leucine and isoleucine biosynthesis
0.555902
[0.53834, 0.57347]
Lysine biosynthesis
1.088779
[1.06971, 1.10785]
Lysine degradation
0.301658
[0.27922, 0.32409]
Tyrosine metabolism
0.514109
[0.49698, 0.53123]
Phenylalanine metabolism
0.354766
[0.34199, 0.36754]
Phenylalanine, tyrosine and tryptophan biosynthesis
1.140863
[1.10969, 1.17203]
M
W
D
N
No
Yes
M
Cereal type
D
CP content
No
Sodium butyrate

Biosynthesis of other secondary metabolism

Penicillin and cephalosporin biosynthesis

Term	Estimate	Std Error	t Ratio	Prob>|t|
Cereal type[M]	-0.000176	0.000999	-0.18	0.8605
CP content[D]	-0.00215	0.000999	-2.15	0.0347*
Cereal type[M]*CP content[D]	0.0007535	0.000999	0.75	0.4531
Sodium butyrate[No]	-0.000696	0.000999	-0.70	0.4880
Cereal type[M]*Sodium butyrate[No]	0.0006935	0.000999	0.69	0.4897
CP content[D]*Sodium butyrate[No]	0.0002784	0.000999	0.28	0.7812
Cereal type[M]*CP content[D]*Sodium butyrate[No]	-0.001038	0.000999	-1.04	0.3023

Novobiocin biosynthesis

Term	Estimate	Std Error	t Ratio	Prob>|t|
Cereal type[M]	-0.001686	0.000467	-3.61	0.0006*
CP content[D]	-0.000089	0.000467	-0.19	0.8496
Cereal type[M]*CP content[D]	0.0002145	0.000467	0.46	0.6478
Sodium butyrate[No]	-0.001191	0.000467	-2.55	0.0130*
Cereal type[M]*Sodium butyrate[No]	0.0006727	0.000467	1.44	0.1545
CP content[D]*Sodium butyrate[No]	-0.000074	0.000467	-0.16	0.8746
Cereal type[M]*CP content[D]*Sodium butyrate[No]	-8.312e-5	0.000467	-0.18	0.8594

Streptomycin biosynthesis

Term	Estimate	Std Error	t Ratio	Prob>|t|
Cereal type[M]	0.006559	0.003052	2.15	0.0350*
CP content[D]	-0.006082	0.003052	-1.99	0.0501
Cereal type[M]*CP content[D]	0.0043481	0.003052	1.42	0.1586
Sodium butyrate[No]	0.0008381	0.003052	0.27	0.7844
Cereal type[M]*Sodium butyrate[No]	0.0035779	0.003052	1.17	0.2449
CP content[D]*Sodium butyrate[No]	-0.004543	0.003052	-1.49	0.1410
Cereal type[M]*CP content[D]*Sodium butyrate[No]	0.0016874	0.003052	0.55	0.5820

Butirosin and neomycin biosynthesis

Term	Estimate	Std Error	t Ratio	Prob>|t|
Cereal type[M]	-0.001056	0.000301	-3.50	0.0008*
CP content[D]	-0.000766	0.000301	-2.54	0.0132*
Cereal type[M]*CP content[D]	0.0005535	0.000301	1.84	0.0704
Sodium butyrate[No]	-0.000679	0.000301	-2.25	0.0272*
Cereal type[M]*Sodium butyrate[No]	0.0007739	0.000301	2.57	0.0123*
CP content[D]*Sodium butyrate[No]	9.4366e-5	0.000301	0.31	0.7551
Cereal type[M]*CP content[D]*Sodium butyrate[No]	-0.000269	0.000301	-0.89	0.3756

Phenylpropanoid biosynthesis

Term	Estimate	Std Error	t Ratio	Prob>|t|
Cereal type[M]	0.0118637	0.004489	2.64	0.0101*
CP content[D]	-0.006258	0.004489	-1.39	0.1676
Cereal type[M]*CP content[D]	0.0050449	0.004489	1.12	0.2648
Sodium butyrate[No]	0.0031132	0.004489	0.69	0.4902
Cereal type[M]*Sodium butyrate[No]	0.003327	0.004489	0.74	0.4610
CP content[D]*Sodium butyrate[No]	-0.006379	0.004489	-1.42	0.1596
Cereal type[M]*CP content[D]*Sodium butyrate[No]	0.0022616	0.004489	0.50	0.6159

Flavonoid biosynthesis

Term	Estimate	Std Error	t Ratio	Prob>|t|
Cereal type[M]	8.675e-5	4.07e-5	2.13	0.0365*
CP content[D]	-0.000053	4.07e-5	-1.30	0.1970
Cereal type[M]*CP content[D]	0.000064	4.07e-5	1.57	0.1203
Sodium butyrate[No]	-0.000026	4.07e-5	-0.64	0.5250
Cereal type[M]*Sodium butyrate[No]	0.000028	4.07e-5	0.69	0.4937
CP content[D]*Sodium butyrate[No]	-7.25e-6	4.07e-5	-0.18	0.8591
Cereal type[M]*CP content[D]*Sodium butyrate[No]	-3.075e-5	4.07e-5	-0.76	0.4524

Flavone and flavonol biosynthesis

Term	Estimate	Std Error	t Ratio	Prob>|t|
Cereal type[M]	0.0012739	0.000398	3.20	0.0020*
CP content[D]	-0.000394	0.000398	-0.99	0.3250
Cereal type[M]*CP content[D]	0.0003435	0.000398	0.86	0.3911
Sodium butyrate[No]	0.0005505	0.000398	1.38	0.1710
Cereal type[M]*Sodium butyrate[No]	8.6776e-5	0.000398	0.22	0.8280
CP content[D]*Sodium butyrate[No]	-0.000652	0.000398	-1.64	0.1058
Cereal type[M]*CP content[D]*Sodium butyrate[No]	0.0003432	0.000398	0.86	0.3915

Betalain biosynthesis

Term	Estimate	Std Error	t Ratio	Prob>|t|
Cereal type[M]	6.7125e-6	4.643e-6	1.45	0.1526
CP content[D]	-1.014e-5	4.643e-6	-2.18	0.0323*
Cereal type[M]*CP content[D]	4.375e-7	4.643e-6	0.09	0.9252
Sodium butyrate[No]	8.125e-7	4.643e-6	0.18	0.8616
Cereal type[M]*Sodium butyrate[No]	3.0375e-6	4.643e-6	0.65	0.5150
CP content[D]*Sodium butyrate[No]	-1.513e-6	4.643e-6	-0.33	0.7455
Cereal type[M]*CP content[D]*Sodium butyrate[No]	7.1125e-6	4.643e-6	1.53	0.1299

Source	LogWorth	PValue
Cereal type	3.245	0.00057

Source	LogWorth	PValue
Cereal type*Sodium butyrate	1.911	0.01229
Sodium butyrate	1.887	0.01296 ^
CP content	1.880	0.01319
Cereal type*CP content	1.153	0.07036
CP content*Sodium butyrate	0.975	0.10582
Cereal type*CP content*Sodium butyrate	0.886	0.12991

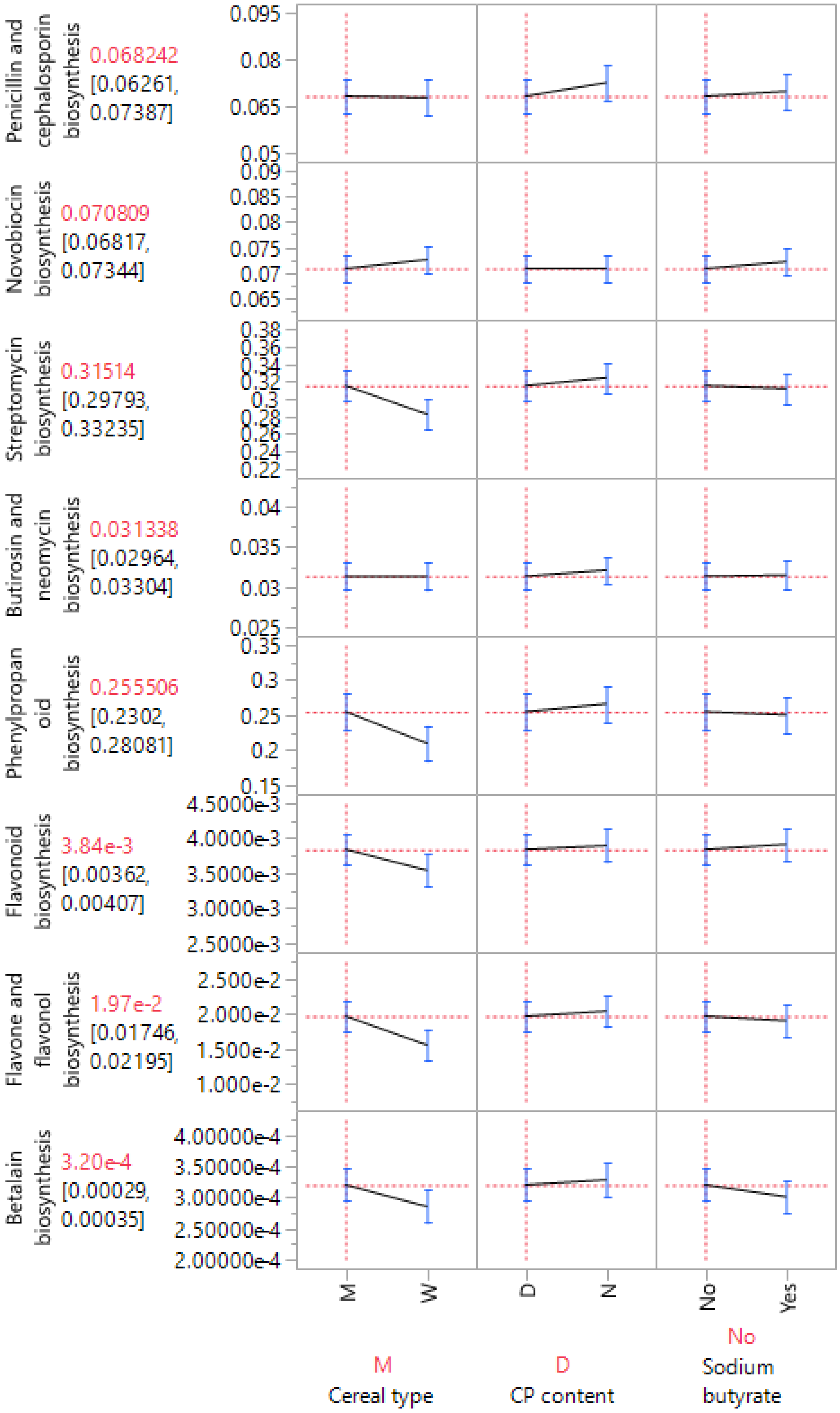

Penicillin and cephalosporin biosynthesis
0.068242
[0.06261, 0.07387]
Novobiocin biosynthesis
0.070809
[0.06817, 0.07344]
Streptomycin biosynthesis
0.31514
[0.29793, 0.33235]
Butirosin and neomycin biosynthesis
0.031338
[0.02964, 0.03304]
Phenylpropanoid biosynthesis
0.255506
[0.2302, 0.28081]
Flavonoid biosynthesis
3.84e-3
[0.00362, 0.00407]
Flavone and flavonol biosynthesis
1.97e-2
[0.01746, 0.02195]
Betalain biosynthesis
3.20e-4
[0.00029, 0.00035]
M
W
D
N
No
Yes
M
Cereal type
D
CP content
No
Sodium butyrate

Carbohydrate metabolism

Glycoisis / gluconeogenesis

Term	Estimate	Std Error	t Ratio	Prob>|t|
Cereal type[M]	-0.019314	0.009355	-2.06	0.0426*
CP content[D]	-0.011278	0.009355	-1.21	0.2320
Cereal type[M]*CP content[D]	0.005131	0.009355	0.55	0.5851
Sodium butyrate[No]	-0.006727	0.009355	-0.72	0.4744
Cereal type[M]*Sodium butyrate[No]	0.0051898	0.009355	0.55	0.5808
CP content[D]*Sodium butyrate[No]	0.0062871	0.009355	0.67	0.5037
Cereal type[M]*CP content[D]*Sodium butyrate[No]	-0.006506	0.009355	-0.70	0.4890

Citrate cyle (TCA)

Term	Estimate	Std Error	t Ratio	Prob>|t|
Cereal type[M]	0.0064875	0.003235	2.01	0.0487*
CP content[D]	-0.005685	0.003235	-1.76	0.0831
Cereal type[M]*CP content[D]	0.0033775	0.003235	1.04	0.2999
Sodium butyrate[No]	0.0025623	0.003235	0.79	0.4309
Cereal type[M]*Sodium butyrate[No]	0.0005723	0.003235	0.18	0.8601
CP content[D]*Sodium butyrate[No]	-0.003914	0.003235	-1.21	0.2302
Cereal type[M]*CP content[D]*Sodium butyrate[No]	0.0015528	0.003235	0.48	0.6327

Pentose phosphate pathway

Term	Estimate	Std Error	t Ratio	Prob>|t|
Cereal type[M]	-0.022243	0.005877	-3.78	0.0003*
CP content[D]	-0.008123	0.005877	-1.38	0.1712
Cereal type[M]*CP content[D]	0.0041261	0.005877	0.70	0.4849
Sodium butyrate[No]	-0.007636	0.005877	-1.30	0.1980
Cereal type[M]*Sodium butyrate[No]	0.0075787	0.005877	1.29	0.2014
CP content[D]*Sodium butyrate[No]	0.0050724	0.005877	0.86	0.3910
Cereal type[M]*CP content[D]*Sodium butyrate[No]	-0.005583	0.005877	-0.95	0.3453

Pentose and glucoronate interconversions

Term	Estimate	Std Error	t Ratio	Prob>|t|
Cereal type[M]	0.0179752	0.006166	2.92	0.0047*
CP content[D]	0.0083158	0.006166	1.35	0.1817
Cereal type[M]*CP content[D]	-0.001516	0.006166	-0.25	0.8064
Sodium butyrate[No]	0.0102886	0.006166	1.67	0.0995
Cereal type[M]*Sodium butyrate[No]	-0.004413	0.006166	-0.72	0.4765
CP content[D]*Sodium butyrate[No]	-0.005837	0.006166	-0.95	0.3470
Cereal type[M]*CP content[D]*Sodium butyrate[No]	0.0029885	0.006166	0.48	0.6294

Fructose and mannose metabolism

Term	Estimate	Std Error	t Ratio	Prob>|t|
Cereal type[M]	0.0587132	0.013952	4.21	<.0001*
CP content[D]	-0.012251	0.013952	-0.88	0.3828
Cereal type[M]*CP content[D]	0.0082387	0.013952	0.59	0.5567
Sodium butyrate[No]	0.0183873	0.013952	1.32	0.1917
Cereal type[M]*Sodium butyrate[No]	-0.002583	0.013952	-0.19	0.8537
CP content[D]*Sodium butyrate[No]	-0.019662	0.013952	-1.41	0.1631
Cereal type[M]*CP content[D]*Sodium butyrate[No]	0.0076289	0.013952	0.55	0.5862

Galactose metabolism

Term	Estimate	Std Error	t Ratio	Prob>|t|
Cereal type[M]	0.009493	0.014933	0.64	0.5270
CP content[D]	-0.030372	0.014933	-2.03	0.0457*
Cereal type[M]*CP content[D]	0.022736	0.014933	1.52	0.1323
Sodium butyrate[No]	0.002911	0.014933	0.19	0.8460
Cereal type[M]*Sodium butyrate[No]	0.0190381	0.014933	1.27	0.2065
CP content[D]*Sodium butyrate[No]	-0.017907	0.014933	-1.20	0.2344
Cereal type[M]*CP content[D]*Sodium butyrate[No]	0.0033653	0.014933	0.23	0.8223

Ascorbate and aldarate metabolism

Term	Estimate	Std Error	t Ratio	Prob>|t|
Cereal type[M]	-0.006488	0.002333	-2.78	0.0069*
CP content[D]	-0.001209	0.002333	-0.52	0.6059
Cereal type[M]*CP content[D]	0.0004391	0.002333	0.19	0.8512
Sodium butyrate[No]	0.0011831	0.002333	0.51	0.6135
Cereal type[M]*Sodium butyrate[No]	-0.001263	0.002333	-0.54	0.5899
CP content[D]*Sodium butyrate[No]	0.0037158	0.002333	1.59	0.1155
Cereal type[M]*CP content[D]*Sodium butyrate[No]	-0.003632	0.002333	-1.56	0.1238

Starch and sucrose metabolism

Term	Estimate	Std Error	t Ratio	Prob>|t|
Cereal type[M]	0.0392548	0.012786	3.07	0.0030*
CP content[D]	0.005075	0.012786	0.40	0.6926
Cereal type[M]*CP content[D]	0.003993	0.012786	0.31	0.7557
Sodium butyrate[No]	-0.01757	0.012786	-1.37	0.1736
Cereal type[M]*Sodium butyrate[No]	0.0160391	0.012786	1.25	0.2137
CP content[D]*Sodium butyrate[No]	-0.020993	0.012786	-1.64	0.1050
Cereal type[M]*CP content[D]*Sodium butyrate[No]	0.0016051	0.012786	0.13	0.9004

Aminosugar and nucleotide sugar metabolism

Term	Estimate	Std Error	t Ratio	Prob>|t|
Cereal type[M]	0.0694364	0.020484	3.39	0.0011*
CP content[D]	-0.028419	0.020484	-1.39	0.1696
Cereal type[M]*CP content[D]	0.0177411	0.020484	0.87	0.3893
Sodium butyrate[No]	0.0145484	0.020484	0.71	0.4799
Cereal type[M]*Sodium butyrate[No]	0.012063	0.020484	0.59	0.5578
CP content[D]*Sodium butyrate[No]	-0.042052	0.020484	-2.05	0.0437*
Cereal type[M]*CP content[D]*Sodium butyrate[No]	0.0207019	0.020484	1.01	0.3156

Pyruvate metabolism

Term	Estimate	Std Error	t Ratio	Prob>|t|
Cereal type[M]	0.0138947	0.005884	2.36	0.0209*
CP content[D]	-0.008508	0.005884	-1.45	0.1525
Cereal type[M]*CP content[D]	0.0031196	0.005884	0.53	0.5976
Sodium butyrate[No]	0.0026414	0.005884	0.45	0.6548
Cereal type[M]*Sodium butyrate[No]	-0.000869	0.005884	-0.15	0.8830
CP content[D]*Sodium butyrate[No]	-0.002518	0.005884	-0.43	0.6700
Cereal type[M]*CP content[D]*Sodium butyrate[No]	0.0015386	0.005884	0.26	0.7945

Glyoxylate and dicarboxylate metabolism

Term	Estimate	Std Error	t Ratio	Prob>|t|
Cereal type[M]	0.0053872	0.003087	1.74	0.0853
CP content[D]	-0.001406	0.003087	-0.46	0.6501
Cereal type[M]*CP content[D]	-0.000596	0.003087	-0.19	0.8475
Sodium butyrate[No]	0.0014689	0.003087	0.48	0.6357
Cereal type[M]*Sodium butyrate[No]	0.0006521	0.003087	0.21	0.8333
CP content[D]*Sodium butyrate[No]	-0.006453	0.003087	-2.09	0.0401*
Cereal type[M]*CP content[D]*Sodium butyrate[No]	0.0084214	0.003087	2.73	0.0080*

C5- Branched dibasic acid metabolism

Term	Estimate	Std Error	t Ratio	Prob>|t|
Cereal type[M]	-0.002694	0.000956	-2.82	0.0062*
CP content[D]	-0.001291	0.000956	-1.35	0.1809
Cereal type[M]*CP content[D]	0.0011602	0.000956	1.21	0.2286
Sodium butyrate[No]	-0.001416	0.000956	-1.48	0.1427
Cereal type[M]*Sodium butyrate[No]	0.0013649	0.000956	1.43	0.1575
CP content[D]*Sodium butyrate[No]	-0.000849	0.000956	-0.89	0.3770
Cereal type[M]*CP content[D]*Sodium butyrate[No]	0.0002364	0.000956	0.25	0.8053

Source	LogWorth	PValue
Cereal type	4.135	0.00007
Cereal type*CP content*Sodium butyrate	2.097	0.00800
CP content*Sodium butyrate	1.397	0.04012 ^
CP content	1.340	0.04566 ^
Sodium butyrate	1.002	0.09953 ^
Cereal type*CP content	0.879	0.13227 ^
Cereal type*Sodium butyrate	0.803	0.15750 ^

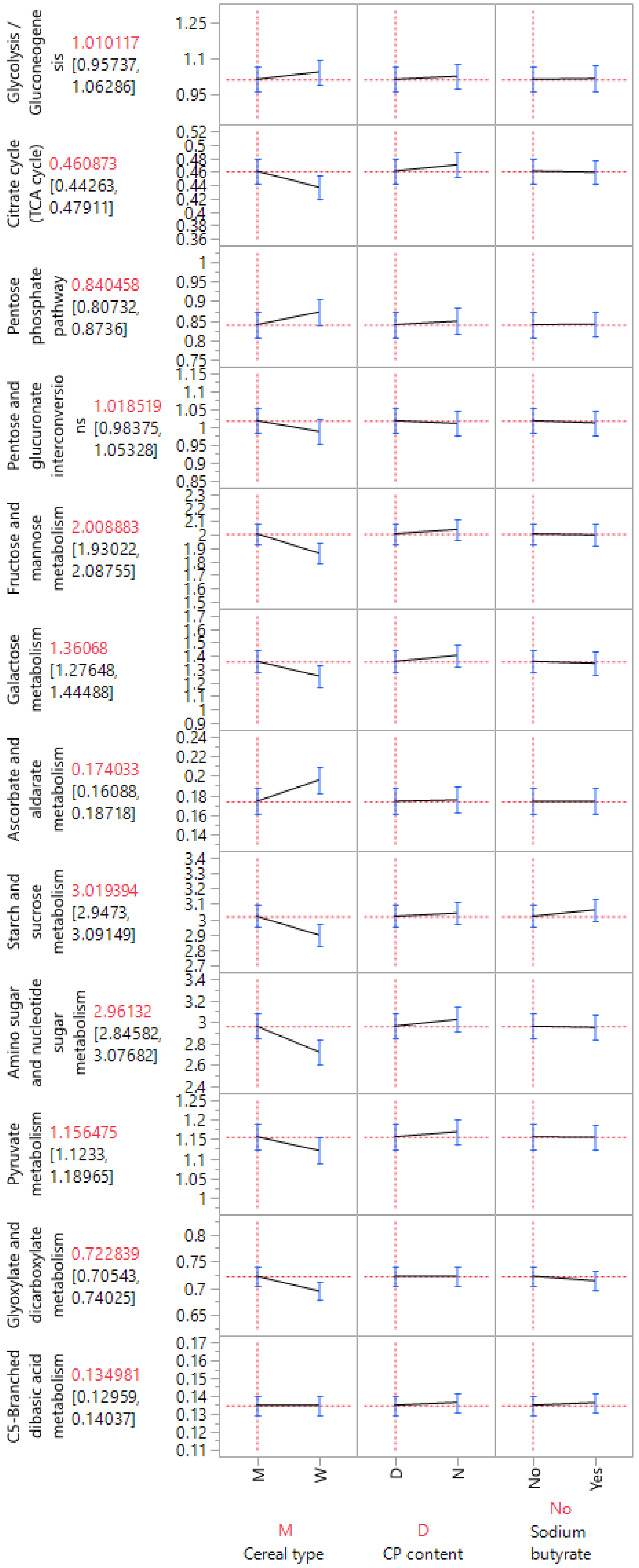
Glycolysis / Gluconeogenesis
1.010117 [0.95737, 1.06286]
Citrate cycle (TCA cycle)
0.460873 [0.44263, 0.47911]
Pentose phosphate pathway
0.840458 [0.80732, 0.8736]
Pentose and glucuronate interconversions
1.018519 [0.98375, 1.05328]
Fructose and mannose metabolism
2.008883 [1.93022, 2.08755]
Galactose metabolism
1.36068 [1.27648, 1.44488]
Ascorbate and aldarate metabolism
0.174033 [0.16088, 0.18718]
Starch and sucrose metabolism
3.019394 [2.9473, 3.09149]
Amino sugar and nucleotide sugar metabolism
2.96132 [2.84582, 3.07682]
Pyruvate metabolism
1.156475 [1.1233, 1.18965]
Glyoxylate and dicarboxylate metabolism
0.722839 [0.70543, 0.74025]
C5-Branched dibasic acid metabolism
0.134981 [0.12959, 0.14037]
M
W
D
N
No
Yes
M
Cereal type
D
CP content
No
Sodium butyrate

Protein export

Term	Estimate	Std Error	t Ratio	Prob>|t|
Cereal type[M]	0.0076379	0.002701	2.83	0.0061*
CP content[D]	-0.004374	0.002701	-1.62	0.1097
Cereal type[M]*CP content[D]	0.0025507	0.002701	0.94	0.3481
Sodium butyrate[No]	-0.00438	0.002701	-1.62	0.1093
Cereal type[M]*Sodium butyrate[No]	0.0036226	0.002701	1.34	0.1841
CP content[D]*Sodium butyrate[No]	-0.003531	0.002701	-1.31	0.1952
Cereal type[M]*CP content[D]*Sodium butyrate[No]	0.0009221	0.002701	0.34	0.7338

Source	LogWorth	PValue
Cereal type	2.217	0.00606
Sodium butyrate	0.962	0.10926
CP content	0.960	0.10970
Cereal type*Sodium butyrate	0.735	0.18406
CP content*Sodium butyrate	0.710	0.19520
Cereal type*CP content	0.458	0.34814
Cereal type*CP content*Sodium butyrate	0.134	0.73381

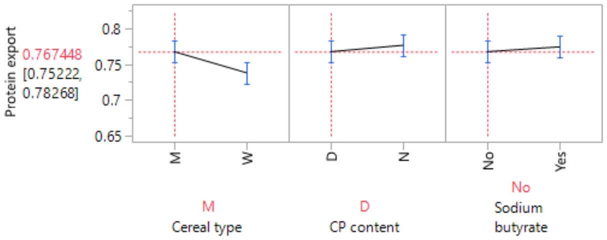

Peptidoglycan biosynthesis

Term	Estimate	Std Error	t Ratio	Prob>|t|
Cereal type[M]	-0.029471	0.010649	-2.77	0.0072*
CP content[D]	-0.006845	0.010649	-0.64	0.5224
Cereal type[M]*CP content[D]	0.0021244	0.010649	0.20	0.8424
Sodium butyrate[No]	-0.028295	0.010649	-2.66	0.0097*
Cereal type[M]*Sodium butyrate[No]	0.0153107	0.010649	1.44	0.1548
CP content[D]*Sodium butyrate[No]	0.0004924	0.010649	0.05	0.9632
Cereal type[M]*CP content[D]*Sodium butyrate[No]	-0.002344	0.010649	-0.22	0.8264

Source	LogWorth	PValue
Cereal type	2.144	0.00718
Sodium butyrate	2.013	0.00970
Cereal type*Sodium butyrate	0.810	0.15483
CP content	0.282	0.52240
Cereal type*CP content*Sodium butyrate	0.083	0.82640
Cereal type*CP content	0.074	0.84244 ^

Source	LogWorth	PValue
CP content*Sodium butyrate	0.016	0.96325 ^

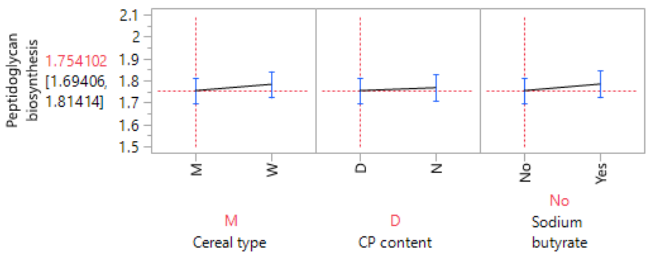

Lipid metabolism

Fatty acid biosynthesis

Term	Estimate	Std Error	t Ratio	Prob>|t|
Cereal type[M]	-0.008392	0.004907	-1.71	0.0916
CP content[D]	-0.013511	0.004907	-2.75	0.0075*
Cereal type[M]*CP content[D]	0.0075179	0.004907	1.53	0.1299
Sodium butyrate[No]	-0.007908	0.004907	-1.61	0.1114
Cereal type[M]*Sodium butyrate[No]	0.0117312	0.004907	2.39	0.0194*
CP content[D]*Sodium butyrate[No]	-0.004368	0.004907	-0.89	0.3764
Cereal type[M]*CP content[D]*Sodium butyrate[No]	0.0012822	0.004907	0.26	0.7946

Fatty acid elongation

Term	Estimate	Std Error	t Ratio	Prob>|t|
Cereal type[M]	-0.000221	5.858e-5	-3.78	0.0003*

Term	Estimate	Std Error	t Ratio	Prob>|t|
CP content[D]	-2.979e-5	5.858e-5	-0.51	0.6127
Cereal type[M]*CP content[D]	3.0763e-5	5.858e-5	0.53	0.6011
Sodium butyrate[No]	-0.000104	5.858e-5	-1.78	0.0787
Cereal type[M]*Sodium butyrate[No]	5.6763e-5	5.858e-5	0.97	0.3358
CP content[D]*Sodium butyrate[No]	2.5037e-5	5.858e-5	0.43	0.6704
Cereal type[M]*CP content[D]*Sodium butyrate[No]	-0.00003	5.858e-5	-0.51	0.6112

Glycerophospholipid metabolism

Term	Estimate	Std Error	t Ratio	Prob>|t|
Cereal type[M]	-0.011912	0.004276	-2.79	0.0068*
CP content[D]	-0.005593	0.004276	-1.31	0.1950
Cereal type[M]*CP content[D]	0.0058586	0.004276	1.37	0.1749
Sodium butyrate[No]	-0.010672	0.004276	-2.50	0.0149*
Cereal type[M]*Sodium butyrate[No]	0.0067772	0.004276	1.59	0.1173
CP content[D]*Sodium butyrate[No]	0.0083139	0.004276	1.94	0.0558
Cereal type[M]*CP content[D]*Sodium butyrate[No]	-0.006144	0.004276	-1.44	0.1551

Ether lipid metabolism

Term	Estimate	Std Error	t Ratio	Prob>|t|
Cereal type[M]	0.0005581	0.000277	2.02	0.0475*

Term	Estimate	Std Error	t Ratio	Prob>|t|
CP content[D]	-0.000416	0.000277	-1.50	0.1373
Cereal type[M]*CP content[D]	0.00017	0.000277	0.61	0.5411
Sodium butyrate[No]	0.0003039	0.000277	1.10	0.2759
Cereal type[M]*Sodium butyrate[No]	-2.546e-5	0.000277	-0.09	0.9270
CP content[D]*Sodium butyrate[No]	-0.000194	0.000277	-0.70	0.4857
Cereal type[M]*CP content[D]*Sodium butyrate[No]	0.0000697	0.000277	0.25	0.8019

Biosynthesis of unsaturated fatty acids

**Term**	**Estimate**	**Std Error**	**t Ratio**	**Prob>|t|**
Cereal type[M]	-0.00147	0.000737	-2.00	0.0498*
CP content[D]	-0.001466	0.000737	-1.99	0.0504
Cereal type[M]*CP content[D]	0.0004212	0.000737	0.57	0.5693
Sodium butyrate[No]	-0.000543	0.000737	-0.74	0.4635
Cereal type[M]*Sodium butyrate[No]	0.0008019	0.000737	1.09	0.2800
CP content[D]*Sodium butyrate[No]	8.612e-7	0.000737	0.00	0.9991
Cereal type[M]*CP content[D]*Sodium butyrate[No]	-0.000216	0.000737	-0.29	0.7705

**Source**	**LogWorth**	**PValue**
Cereal type	3.493	0.00032
CP content	2.127	0.00746
Sodium butyrate	1.828	0.01485
Cereal type*Sodium butyrate	1.711	0.01943
CP content*Sodium butyrate	1.254	0.05575
Cereal type*CP content	0.886	0.12989
Cereal type*CP content*Sodium butyrate	0.810	0.15506

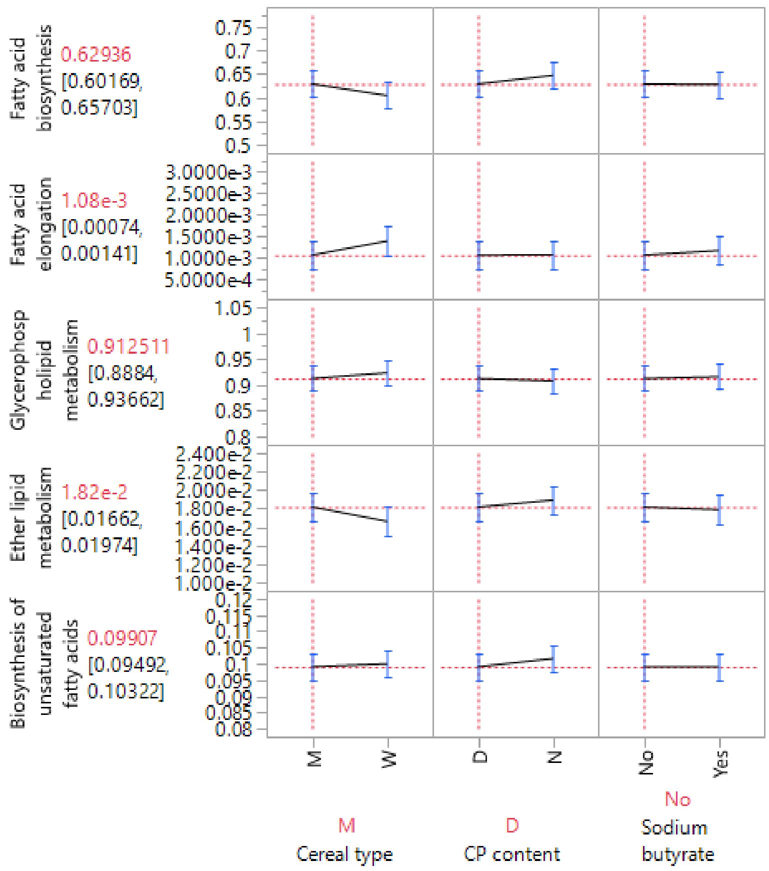
Fatty acid biosynthesis
0.62936
[0.60169, 0.65703]
Fatty acid elongation
1.08e-3
[0.00074, 0.00141]
Glycerophospholipid metabolism
0.912511
[0.8884, 0.93662]
Ether lipid metabolism
1.82e-2
[0.01662, 0.01974]
Biosynthesis of unsaturated fatty acids
0.09907
[0.09492, 0.10322]
M
W
D
N
No
Yes
M
Cereal type
D
CP content
No
Sodium butyrate

Membrane transport

ABC transporters

Term	Estimate	Std Error	t Ratio	Prob>|t|
Cereal type[M]	-0.309404	0.089692	-3.45	0.0009*
CP content[D]	0.1181972	0.089692	1.32	0.1917
Cereal type[M]*CP content[D]	-0.071221	0.089692	-0.79	0.4298
Sodium butyrate[No]	-0.128479	0.089692	-1.43	0.1563
Cereal type[M]*Sodium butyrate[No]	0.0012544	0.089692	0.01	0.9889
CP content[D]*Sodium butyrate[No]	0.103677	0.089692	1.16	0.2515
Cereal type[M]*CP content[D]*Sodium butyrate[No]	-0.052495	0.089692	-0.59	0.5602

Phosphotransferase system

Term	Estimate	Std Error	t Ratio	Prob>|t|
Cereal type[M]	-0.055475	0.022369	-2.48	0.0155*
CP content[D]	-0.013283	0.022369	-0.59	0.5545
Cereal type[M]*CP content[D]	0.0091478	0.022369	0.41	0.6838
Sodium butyrate[No]	-0.019711	0.022369	-0.88	0.3812
Cereal type[M]*Sodium butyrate[No]	0.0052792	0.022369	0.24	0.8141
CP content[D]*Sodium butyrate[No]	0.027703	0.022369	1.24	0.2196
Cereal type[M]*CP content[D]*Sodium butyrate[No]	-0.02485	0.022369	-1.11	0.2703

Source	LogWorth	PValue
Cereal type	3.026	0.00094
Sodium butyrate	0.806	0.15634
CP content	0.717	0.19174
CP content*Sodium butyrate	0.658	0.21957
Cereal type*CP content*Sodium butyrate	0.568	0.27030
Cereal type*CP content	0.367	0.42977 ^
Cereal type*Sodium butyrate	0.089	0.81410 ^

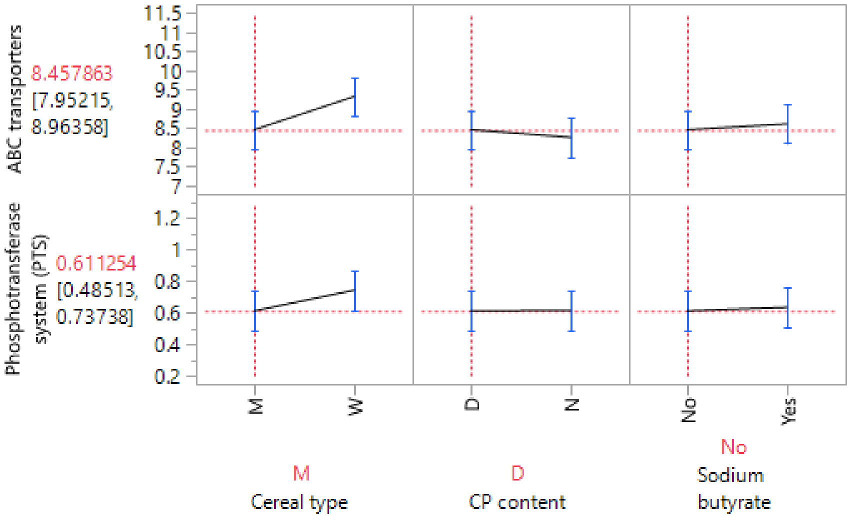

Supplement: Supplementary file 1 [file Data_Sheet_1.pdf]
